# Nodule-associated diazotrophic community succession is driven by developmental phases combined with microhabitat of *Sophora davidii*

**DOI:** 10.3389/fmicb.2022.1078208

**Published:** 2022-12-01

**Authors:** Jiamin Ai, Tianfei Yu, Xiaodong Liu, Yingying Jiang, Ziwei Hao, Xiaoyu Zhao, Entao Wang, Zhenshan Deng

**Affiliations:** ^1^College of Life Sciences, Yan’an University, Yan’an, China; ^2^, Departamento de Microbiología, Escuela Nacional de Ciencias Biológicas, Instituto Politécnico Nacional, Mexico City, Mexico

**Keywords:** *Sophora davidii*, root nodules, diazotrophic community, *nif*H, rhizobia, succession, interaction

## Abstract

Nodule-associated nitrogen-fixing microorganisms (diazotrophs) residing in legume root nodules, and they have the potential to enhance legume survival. However, the succession characteristics and mechanisms of leguminous diazotrophic communities remain largely unexplored. We performed a high-throughput *nif*H amplicon sequencing with samples of root nodules and soil in the three developmental phases (young nodules, active nodules and senescent nodules) of the *Sophora davidii* (Franch.) Skeels root nodules, aiming to investigate the dynamics of nodule-endophytic diazotrophs during three developmental phases of root nodules. The results demonstrated the presence of diverse diazotrophic bacteria and successional community shifting dominated by *Mesorhizobium* and *Bradyrhizobium* inside the nodule according to the nodule development. The relative abundance decreased for *Mesorhizobium*, while decreased first and then increased for *Bradyrhizobium* in nodule development from young to active to senescent. Additionally, strains *M. amorphae* BT-30 and *B. diazoefficiens* B-26 were isolated and selected to test the interaction between them in co-cultured conditions. Under co-culture conditions: *B. diazoefficiens* B-26 significantly inhibited the growth of *M. amorphae* BT-30. Intriguingly, growth of *B. diazoefficiens* B-26 was significantly promoted by co’culture with *M. amorphae* BT-30 and could utilize some carbon and nitrogen sources that *M. amorphae* BT-30 could not. Additionally, the composition of microbial community varied in root nodules, in rhizosphere and in bulk soil. Collectively, our study highlights that developmental phases of nodules and the host microhabitat were the key driving factors for the succession of nodule-associated diazotrophic community.

## Introduction

One of the unique characteristics of legumes is the formation of root and/or stem nodules with the soil bacteria so called rhizobia, which are capable of reducing the atmospheric nitrogen (N_2_) inside the nodules and offer ammonia for their assimilation and for their host plants. Biological N_2_ fixation (BNF), or reduction of N_2_ into ammonia by diazotrophs using nitrogenase (Yin et al., 2018), by the symbiosis between legumes and rhizobia plays a significant role in the global N cycle and is responsible for fixation of as much as 100 Tg N per year on a global scale, contributing more than 97% of the N input in natural terrestrial ecosystems (Kumar et al., 2017). In nature, BNF is performed only by some prokaryotes that can be plant symbionts or free-living diazotrophs. Diazotrophs are highly diverse and widely distributed in different ecosystems, e.g., soil, oceans (Cheung et al., 2021), and the forefields of receding glaciers (Duc et al., 2009), and are directly related to the ecosystem function. Among them, soil harbors the most diverse diazotrophic microbial communities, with Proteobacteria and Cyanobacteria as the most abundant members (Gaby and Buckley, 2011). Many factors have been reported to affect the structure of diazotrophic community, such as temperature (Feng et al., 2019) and precipitation (Yeager et al., 2012), plant species richness (Sheng et al., 2019), soil type and pH (Zhang et al., 2018; Han et al., 2019), agricultural activities such as nitrogen fertilization (Chen et al., 2021). In addition, some seasonal factors and growth cycle of the host plant (Pereira et al., 2013) also regulated the diazotrophic communities that caused a clear seasonal succession of these microbes in some environments (Zilius et al., 2021).

Although rhizobia are predominant in nodules of the legume plants, successful infection by rhizobia depends not only on the competitive ability of different rhizobial species but also the ability of rhizobia to cope with various fluctuating environmental factors. For example, *Bradyrhizobium* strains are predominant in nodules of the soybean in acid soil, while *Sinorhizobium* strains are predominant in soybean nodules in alkaline-saline soil (Zhang et al., 2020). Thus, both the environmental and genetic factors working together largely determine the diazotrophic microbiome assembly inside the nodules. Previous studies have reported that the microbiomes of rhizosphere are a part of the bulk soil community derived from root selection (Philippot et al., 2013; Mendes et al., 2014). Therefore, the microbial community composition in the bulk soil is distinctive from that in the root environment compartments (Reinhold-Hurek et al., 2015), which revealed a decrease in diversity with proximity to the root (Bulgarelli et al., 2013; Donn et al., 2015). For example, the alpha diversities of the microbial communities decreased from bulk soil to rhizosphere to root nodule (Xiao et al., 2017). Microbial communities can be strongly structured by plant compartments, and this is potentially case for legume nodules. Nodules represent a truly unique environment dominated by single microbial taxon (i.e., rhizobia), creating the opportunity for manifold bacteria–bacteria interactions, which in turn contribute to the overall community structure. Thus, nodule-associated microbiomes include hundreds of cohabitant bacteria. However, the mechanisms that underlie this self-organization, microbial interactions within the nodule and seasonal succession among bacteria remain largely elusive.

Despite considerable efforts toward elucidating the composition and the driving force for shifting in community composition of root nodule bacteria, the rules that govern their succession are still not fully understood. The relative simplicity of the nodule microbiome and the observation that members of the microbiome with potential for specialized metabolism make the nodule an attractive system for exploring microbial interactions and the ecological roles of specialized metabolites *in situ* (Hansen et al., 2020). In a recent study, Schäfer et al. (2022) found bacterial interactions in the phyllosphere microbiota and reported that 90% of the identified interactions in planta were negative, and community changes could be largely explained by binary interactions, demonstrating that direct bacteria–bacteria interactions were responsible for the population shifting. However, the mechanism for enrichment process and succession of the nodule-associated diazotrophic microbiota across the lifetime of individual nodules still remains poorly characterized, and research on what drives the interactions in microbial communities is still in its infancy.

*Sophora davidii* (Franch.) Skeels is a drought resistant perennial leguminous shrub, growing in harsh habitats such as river valley dunes, bushes on hillside roads, and the loess hilly region in China, where present fragile ecological environment with serious soil erosion (Luo et al., 2021). *Sophora davidii* has important ecological value in the succession of plant communities in arid regions, maintenance of species diversity, soil improvement and soil erosion control. However, the study on diazotrophic communities associated with this plant is underexplored, except a study on diversity of *S. davidii* rhizobia (Cao et al., 2021). Therefore, in this study, we performed amplification and sequencing of *nif*H genes in nodules and rhizosphere soil of *S. davidii* along the nodule development phases, in comparison with that in bulk soils. In addition, two rhizobial strains representing the diazotrophs with significant change in abundance over the life cycle of individual nodules were isolated and used for exploring their roles on nodulation of *S. davidii*. The aims of this study were: (i) to clarify the shifts in nodule microbiome along with the developmental phases of root nodules and to estimate the ecological drivers for the succession of bacterial communities in the root nodule, rhizosphere soil and bulk soil; (ii) to estimate the members in nodule microbiome that strongly interact through cooperation or competition; and (iii) to analyze the key driver for structure shifting of the nodule-associated diazotrophic microbiota in different developmental phases of root nodule.

## Materials and methods

### Sampling sites and sample collection

The sampling field is located in Baota District (36°37′15″N, 109°22′8″E), Yan’an City, Shaanxi Province, China, located in the loess hilly region, along the monsoon boundary zone in East Asia, with an arid but unstable climate and a clear trend toward a warmer and drier climate. The sampling field has an annual average temperature of 10.0°C and a mean annual precipitation of 527 mm. It is an important distribution area for China’s fragile ecological environment with a significant regional impact of global change, and also presents serious soil erosion.

**Figure 1:**
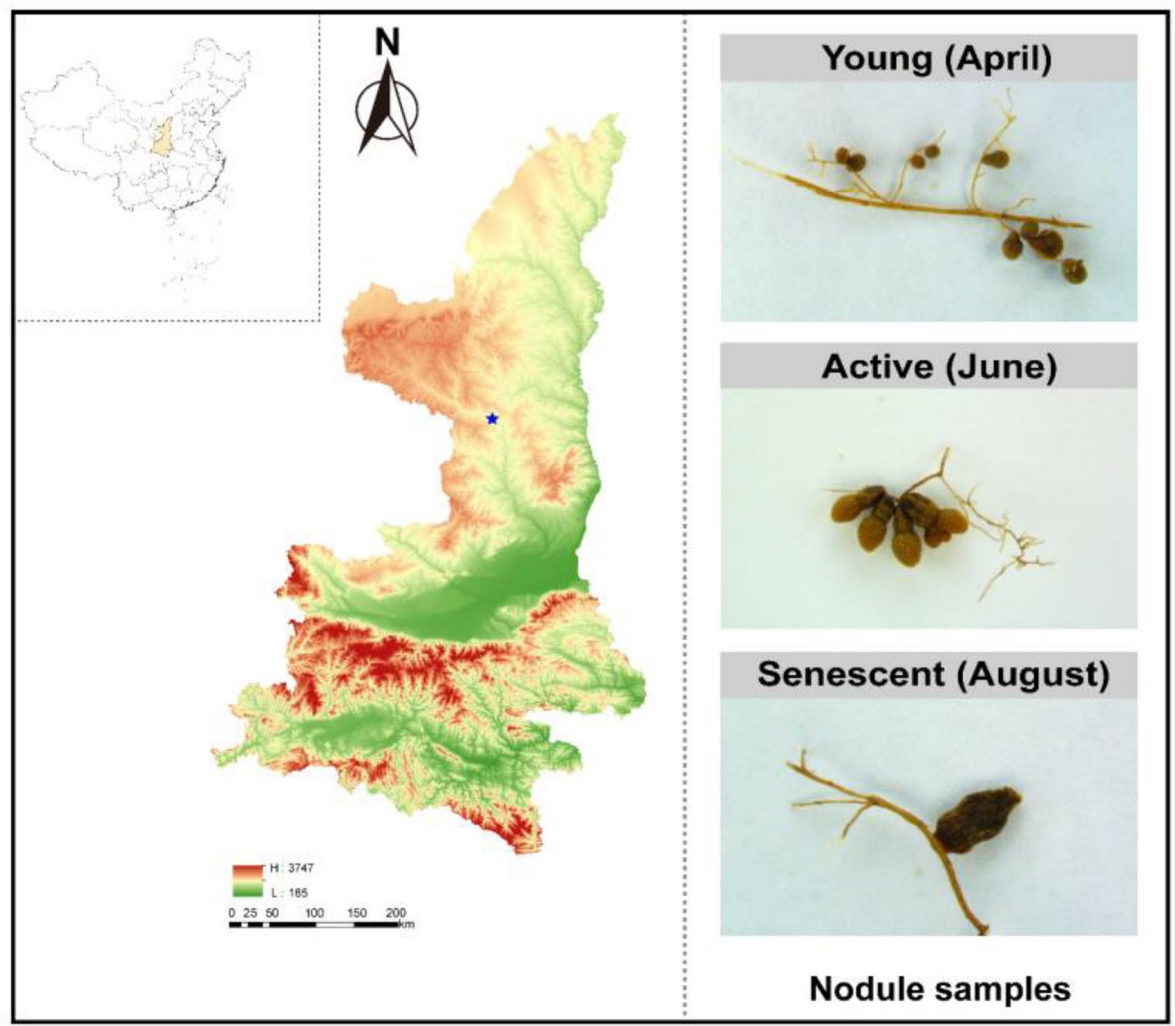
Sampling map. Maps showing elevation, and other geographic properties (left). The collected nodules samples from three phases: young phases (April 2021), active phases (June 2021) and senescent phases (August 2021) (right).

In 2021, samples were taken in three developmental phases of *S. davidii* nodules (Hansen et al., 2020): young nodules (small and light brown without BFN activity) on April 9, active nodules (red-brown/red with BFN activity) on June 20, and senescent nodules (brown coloration without BFN activity) on August 20 (Figure 1). The pink/red color in active nodules is due to the presence of leghemoglobin, whereas the brown color present during senescence is due to the degradation of the heme group associated with leghemoglobin (Van de Velde et al., 2006). To address whether or not the nodules and soil microbiomes differed across these three phases, we collected nodules and rhizosphere soil from established wild-grown *S. davidii,* while the adjacent bulk soils were also sampled. Three sites were randomly selected from the sampling field as biological replicates, and three *S. davidii* plants at the same growth stage were selected from each site to reduce the bias among different plants. The nodules and rhizosphere soils were sampled by digging up a part of the roots as mentioned elsewhere (Xiao et al., 2017). The root systems of plants were carefully removed from the soil, rhizosphere soil <2 mm from the root was excised with a scalpel and root nodules were detached (Nuccio et al., 2020). The sampled plants were kept (replanted) *in situ* for further growth and were marked to ensure the samples always taking from the same plant at the three phases. The nodules and rhizosphere samples collected from each *S. davidii* plant in the same site were blended as one sample. Since the composition of diazotrophic community might be also affected by the root compartments, as revealed for the whole microbiome (Xiao et al., 2017), a surface layer of 5–20 cm of soil was taken at the same time, from the rootless area next to the plant as a bulk soil.

Placed in sterile sampling bags, the samples of nodules, rhizosphere soils, and half of the bulk soils were transported on ice to the laboratory immediately and stored at −80°C for the subsequent extraction of metagenomic DNAs. The other half of the soil samples was air dried and sieved through a 2 mm mesh screen for physicochemical analyses.

### Soil chemical properties

Seven soil physiochemical properties (SCPs), i.e., soil pH, total nitrogen (TN), available phosphorus (AP), available potassium (AK), soil organic matter (OM), nitrate nitrogen (NO_3_^−^-N) and ammonium nitrogen (NH_4_^+^-N) were determined. Soil pH was measured in a soil: water (1:2.5) extract with a pH meter. Soil TN were measured using the Kjeldahl method. Soil AP was determined by the NaHCO_3_ extraction method (Wang et al., 2020). Soil AK was determined with the NH_4_OAC extraction method by flame photometer. Soil OM was determined with by K_2_Cr_2_O_4_ volumetric method. Soil NO_3_^−^N and NH_4_^+^-N were determined by colorimetry and Nessler’s reagent (Liu et al., 2020). Detailed methods for determination of soil physiochemical properties and instruments are shown in Supplementary Table S1.

### DNA extraction, sequencing, and analysis

Metagenomic DNA was extracted from 0.5 g fresh rhizosphere or bulk soil using a TIANamp Soil DNA Kit (TIANGEN Biotech, Beijing, China) according to the manufacturer’s instructions. Nodules were sterilized and decontaminated of surface DNA prior to microbial DNA extraction (Sharaf et al., 2019), and then DNA was extracted from the sterile nodules also using the TIANamp Soil DNA Kit (TIANGEN Biotech, Beijing, China) according to the manufacturer’s guidelines. The concentration and purity of DNA extracts were monitored by electrophoresis in 1% (w/v) agarose gels. The DNA extract was diluted to 1 ng/μl with sterile water and were stored at −20°C for further analysis.

Using diluted genomic DNA as the template, the *nif*H fragments were amplified with primers *nif*H-F (5′-AAAGGYGGWATCGGYAARTCCACCAC-3′) and *nif*H-R (5′-TTGTTSGCSGCRTACATSGCCATCAT-3′; Rösch et al., 2002). The PCR reactions were carried out with 15 μl of Phusion^®^ High -Fidelity PCR Master Mix (New England Biolabs, United States); 2 μM of both the forward and reverse primers, and about 10 ng template DNA. Thermal cycling consisted of initial denaturation at 98°C for 1 min, followed by 30 cycles of denaturation at 98°C for 10 s, annealing at 50°C for 30 s, and elongation at 72°C for 30 s. Finally, 72°C for 5 min. PCR products from the same sample were mixed in equal density ratios. Then, mixture of the PCR products was purified with Qiagen Gel Extraction Kit (Qiagen, Germany). Sequencing libraries were generated using TruSeq^®^ DNA PCR-Free Sample Preparation Kit (Illumina, United States) following manufacturer’s recommendations and index codes were added. The library quality was assessed on the Qubit@ 2.0 Fluorometer (Thermo Scientific) and Agilent Bioanalyzer 2100 system. At last, the library was sequenced on an Illumina NovaSeq platform and 250 bp paired-end reads were generated.

According to the Barcode sequence and PCR amplification primer sequence, the *nif*H gene sequences for each sample were separated from the offline data. After Barcode and primer sequences were truncated, the reads of each sample were spliced using FLASH 1.2.7 to obtain the original Tag data (Magoč and Salzberg, 2011), and then Qiime 1.9.1 was used for quality control (Caporaso et al., 2010) to remove the chimera and finally obtain the effective data (Haas et al., 2011). The Uparse algorithm was used to cluster the effective Tags of all samples with 97% consistency to form the optional taxonomy units (OTUs; Edgar, 2013). Sequence with the highest frequency in each OTU was selected as the representative to compare with the Nucleotide Sequence Database for obtaining the species annotation.

Alpha diversity was applied for analyzing complexity of species diversity for a sample through 4 indices, including Chao1, Shannon, Simpson, and ACE, which were calculated with QIIME 1.7.0 and displayed with R software 2.15.3. Beta diversity was calculated by QIIME software 1.9.1. Principal component analysis (PCA) was displayed by R software 2.15.3. Canonical correlation analysis (CCA) was used for the sorting analysis performed by the CCA functions in the vegan package R software 2.15.3. Linear discriminant analysis (LDA) was used to analyze potential marker microorganisms with an LDA score of 2.5 (Segata et al., 2011).

### Rhizobial isolation and identification

The disinfection of root nodules was performed with a standard protocol (Kamicker Barbara and Brill Winston, 1986). Surface-sterilized root nodules were crushed and streaked on yeast mannitol agar (YMA; Graham, 1969) plates with sterile forceps and then incubated at 28°C for 5–7 days. The isolated colonies were purified by repeatedly cross-streaking on the same medium. Finally, the purified isolates were stored at −80°C in YM broth supplied with 30% (v/v) of glycerol. To identify the isolates, genomic DNA was extracted and 16S rRNA gene was amplified and sequenced as mentioned above. A homology comparison of the acquired sequences was conducted by Blast research using GenBank database for getting the related reference sequences, and the phylogenetic tree was constructed using the MEGA. 5.0 software with Neighbor-joining method (Tamura et al., 2011). Then matched bacterial isolates with OUT representative sequences by SnapGene and BLAST in NCBI. NCBI accession numbers of *Mesorhizobium amorphae* BT-30 and *Bradyrhizobium diazoefficiens* B-26 16S rRNA genes were ON598668 and ON598635, respectively. In addition, the *nif*H gene of the isolates were sequenced. The *nif*H gene sequences of the isolates and OTUs sequences were imported into SnapGene and match them. All OUT representative sequences were deposited into the NCBI Sequence Read Archive (SRA) database under the accession number SUB11740897.

### Resource utilization patterns of the bacterial isolates

Using YM broth as the basal medium, the isolates were tested for growth with different nitrogen sources (sodium nitrate, potassium nitrate, calcium nitrate, ammonium sulfate, ammonium chloride, ammonium phosphate monobasic, ammonium nitrate, yeast extract, casein acid hydrolysate and urea at the concentration of 3 g/L, mannitol as C-source, pH 7.0); with different carbon sources (D-mannitol, D-glucose, D-xylose, starch soluble, sucrose, citric acid monohydrate, D-fructose, inositol, L-rhamnose, D-arabinose at the concentration of 10 g/L, yeast extract as N source, pH 7.0); at pH (4, 5, 6, 7, 8, 9, 10, and 11); and at salt concentrations (0, 5, 10, 20, 30, 40, 50 and 60 g/L, pH 7.0). All tests were cultured at 28°C with agitation of 170 rpm for 4 day, and then bacterial growth was determined by measuring the optical density (OD) at 600 nm by microplate reader platform (800TS, BioTeK, United States; Mahler et al., 2000).

### Interaction assay

#### Growth interaction on YMA

The isolates identified as *Bradyrhizobium diazoefficiens* B-26 and *Mesorhizobium amorphae* BT-30 were incubated separately for 4–6 days at 28°C in YM broth, and the OD_600_ of the cultures were adjusted to 1.0. Next, aliquot of 1.5 μl suspension of each strain was spotted at 30 mm (control),15 mm and 10 mm distance from each other on YMA plates (Han et al., 2020). The plates were incubated at 28°C for 6–8 days. Each assay was carried out in three replicates. Images were captured using an automatic colony analyzer (Czone G6T, Shineso, Hangzhou, China), and the colony diameters were measured for presenting the interaction. Each assay was carried out in three replicates.

#### Production of exopolysaccharides

EPS production might be used as an indicator for promotion or inhibition of growth in the interaction between the bacterial strains (Ghosh and Maiti, 2016). In this study, the colonies obtained in the growth interaction were collected from YMA plates, suspended in sterile distilled water, washed with sterile distilled water twice by vortexing vigorously at room temperature (25°C) for 5 min, and centrifuged (12,000 rpm) for 10 min at 4°C. The supernatant was transferred into another tube for EPS isolation, while the cell pellet was dried and weighed. EPS was precipitated from the supernatant (20 ml) with 96% (v/v) cold ethanol at volumetric ratio of 1:4 (supernatant/ethanol). Then, EPS was collected by centrifugation (11,000 rpm) at 4°C for 30 min, and the pellet was washed three times with 100% (v/v) ethanol. After the ethanol was evaporated, the EPS was dissolved in sterile deionized water, and total EPS content was measured using the phenol-sulfuric method (Albalasmeh et al., 2013).

#### Measurement of growth curves

For this analysis, strains *B. diazoefficiens* B-26 and *M. amorphae* BT-30 were cultured separately at 28°C with shaking of 150 rpm in YM broth, and the OD_600_ of the cultures were adjusted to 1.0. For growth curves of each strain, the cultures mentioned above were inoculated separately into the YM broth at a ratio of 1% (v/v). The growth curves of *M. amorphae* BT-30 under the influence of metabolites of *B. diazoefficiens* B-26. Firstly, culture *B. diazoefficiens* B-26 overnight, then centrifuged at 12,000 rpm. For 5 min and filtered through a 0.22 μm membrane filter to remove bacterial thallus. Then, culture of *M. amorphae* BT-30 was introduced into the supernatant of *B. diazoefficiens* B-26 at a ratio of 1% (v/v). The growth curves of *B. diazoefficiens* B-26 under the influence of metabolites of *M. amorphae* BT-30 was taken the same way. In both cases, the cultures were incubated at 28°C with shaking of 150 rpm, and OD_600_ was measured every 12 h up to 160 h. The specific cell growth rate (μx) was calculated using the slope of the semi-logarithmic curve of cell density versus time according to Eqs (Feng et al., 2021).

### Statistical analyses

The data shown in the corresponding figures and tables represent the means values and the standard deviations. Statistical analysis was performed with SPSS 22.0. One-way ANOVA was used to compare the value of α-indices. The significance of the production of EPS, colony diameter and plant growth promoting effects of the microorganisms between treatment and control groups were calculated by *T*-test. The value of significance (*p*-value) was adjusted by multiple comparison tests with the Benjamin-Hochberg method. Other statistics analyses were performed in ORIGIN 2020.

## Results

### Soil physicochemical properties

Soil physiochemical properties varied considerable with time variation (Supplementary Figure S1). The contents of AP and NH_4_^+^-N increased with time variation in rhizosphere and bulk soil. The content of AK presented a decreasing trend over time in rhizosphere, but showed the highest value in young phase and the lowest in active phase for bulk soil. The contents of OM in rhizosphere and bulk soil both peaked in the active phase. The contents of NO_3_^−^-N increased significantly (*p* < 0.001) in the senescent phase for rhizosphere, but peaked in young phase for bulk soil. TN content reached the highest in active phase in rhizosphere and gradual increase over time for bulk soil. The pH values in rhizosphere and bulk soil all increased first and then decreased, but were always weakly alkaline. These results show that the physiochemical properties of soil are not static, but changed with time. Moreover, some soil physiochemical properties in rhizosphere soil and bulk soil showed different trends.

### Summary of *nif*H gene sequencing

We obtained a total of 2,248,410 raw reads by Illumina MiSeq high-throughput sequencing. After quality filtering, trimming, and assigning reads to the samples, 2,221,988 high-quality reads were recovered in the dataset, representing 11,076 operational taxonomic units (OTUs) based on 97% sequence identity across all samples. The rarefaction curves of all samples were plateaued (Supplementary Figure S2), suggesting that the sequencing depth was sufficient.

### Alpha diversity of diazotrophic communities (*nif*H)

**Figure 2:**
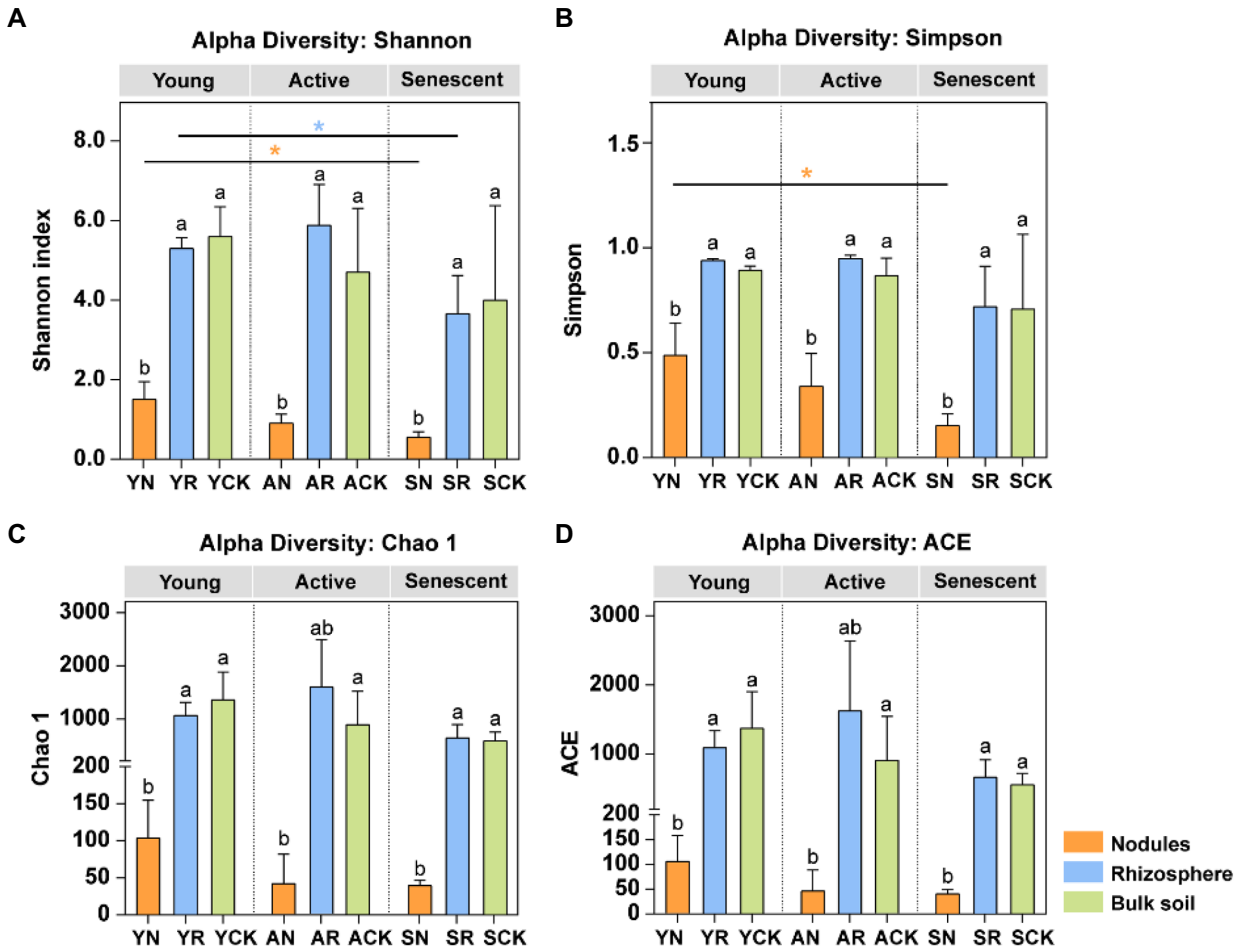
Richness and diversity of the diazotrophic community of the various compartments and phases. **(A)** Shannon index; **(B)** Simpson index; **(C)** Chao 1; **(D)** ACE. Lowercase letters above the bars indicate significant differences (*p* < 0.05) between the different compartments in three phases, respectively (One-way ANOVA test). Asterisks indicate significant differences between three phases in the same compartment (Tukey test). *, *p* < 0.05; **, *p* < 0.01; ***, *p* < 0.001.

The bacterial diversity of diazotrophic communities was estimated by Shannon index, Simpson index, Chao 1, and the ACE, respectively (Figure 2). We observed the diazotrophic diversity (Shannon index, Simpson index, Chao 1, and the ACE) in rhizosphere was higher than that in the bulk soil in active phase. Moreover, the diazotrophic diversities in the nodules were significantly lower than those in the rhizosphere and bulk soil, regardless of the developmental phases (One-way ANOVA test, *p* < 0.05). The diazotrophic diversities (Shannon index and Simpson index) of the nodules in young phase were significantly higher than those in senescent phase (Tukey test, *p* < 0.05). The diazotrophic diversity (Shannon index) of the rhizosphere in young phase was significantly higher than those in senescent phase (Tukey test, *p* < 0.05). In summary, the diversity of diazotrophic communities in rhizosphere and bulk soil was much higher than that in nodules, and rhizosphere soil had the highest diazotrophic diversity in active phase.

### Diazotrophic community profiling across root nodule development

At the phylum level, diazotrophs in Proteobacteria, Actinobacteria and one unclassified phylum had a high relative abundance in all samples. Among them, *nif*H corresponding to the Proteobacteria was the dominant phyla (except the unclassified phylum) in all samples, but showed different relative abundances in three phases and different compartments (Supplementary Figure S3A). In nodules, *nif*H of Proteobacteria was absolutely dominant in all the three phases, and its relative abundance ranged from 98.99% (young phase) to 99.86% (active phase). In rhizosphere, the relative abundances of Proteobacteria diazotroph ranged from 31.03% to 60.13%, and increased gradually over time. In bulk soil, the relative abundances of Proteobacteria diazotroph showed different variation patterns, being the highest in senescent phase (44.96%) and the lowest in active phase (27.80%).

**Figure 3:**
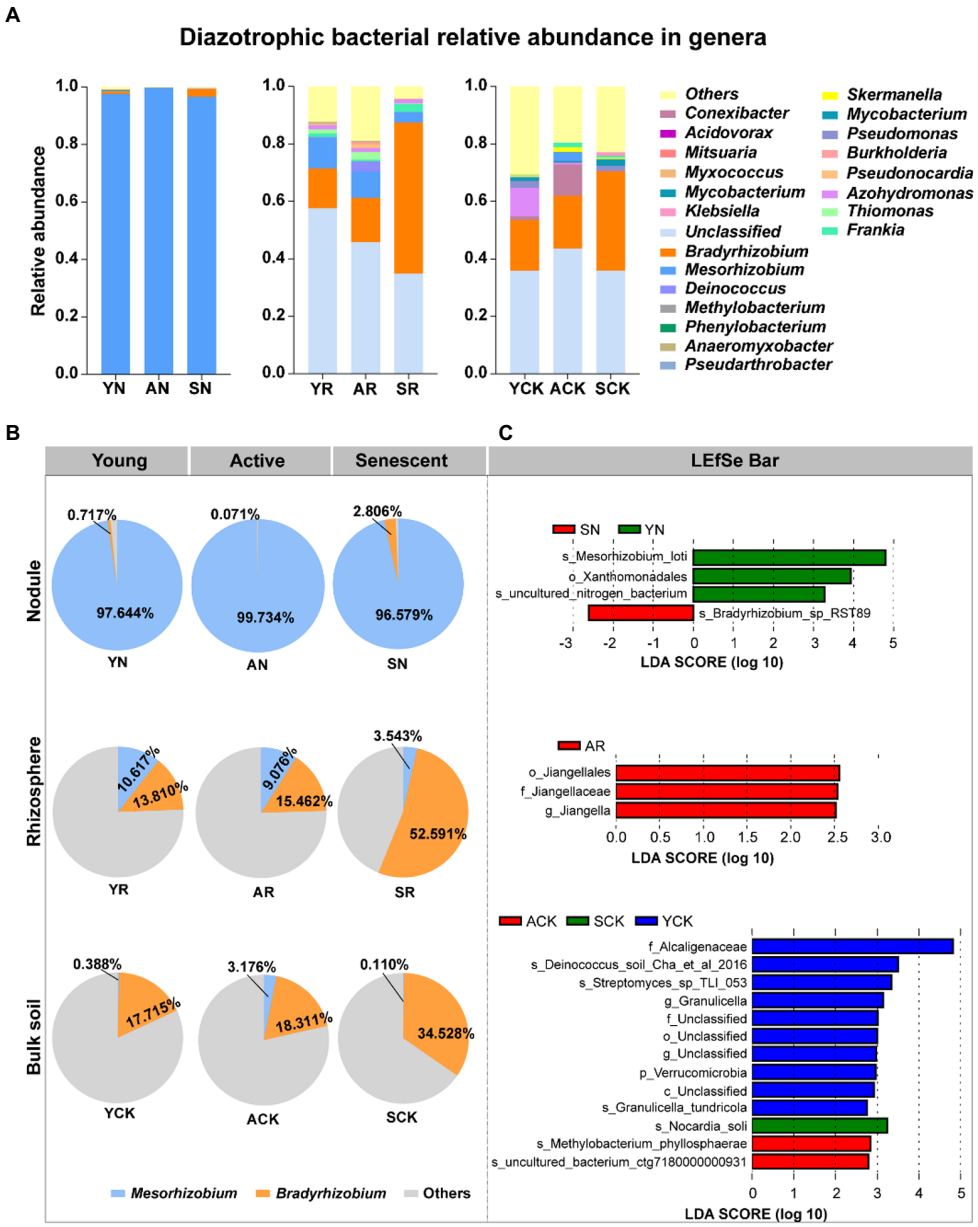
Comparison of the major bacterial composition. **(A)** The distribution of communities at genus level. **(B)** Proportion of relative abundances of *Mesorhizobium* and *Bradyrhizobium* in nine samples. **(C)** Differentially abundant diazotroph taxa in nodules, rhizosphere soil and bulk soil. Detected using linear discriminant analysis effect size analysis.

The species accumulation diagram at the genus level (Figure 3A) and Venn diagram (Supplementary Figure S3B) showed overlap and difference in diazotrophic microbial communities among the samples. It is noteworthy to mention that *Mesorhizobium* and *Bradyrhizobium* are dominant genera in all samples irrespective of the phases, but their relative abundances present different trends (Figure 3B). In nodules, *Mesorhizobium* was the absolutely dominant (99.73%–96.58%) in all the three phases, and its relative abundance showed no significant difference in different phases. Although the relative abundance of *Bradyrhizobium* in nodules was low, significant variations in it were observed among the three phases: from 0.72% (young phase) decreased to 0.07% (active phase), and then increased to 2.81% in senescent phase. In rhizosphere, the relative abundance of *Mesorhizobium* ranged from 10.62% to 3.54%, with a decreasing trend over time. While *Bradyrhizobium* showed the opposite trend and presented relative abundance from 13.81% to 52.59%, much greater than that of *Mesorhizobium*. In bulk soil, the relative abundance of *Mesorhizobium* was increased first and then decreased, ranging from 0.11% (senescent phase) to 3.18% (active phase). The relative abundance of *Bradyrhizobium* showed an increased trend over time, ranging from 17.72% (young phase) to 34.53% (senescent phase). Compared with soil samples, *Mesorhizobium* was significantly enriched in nodules, but there was no significant difference between rhizosphere and bulk soil (Supplementary Tables S2, S3). In summary, the diazotrophic microbial communities in nodules, rhizosphere soil and bulk soil had succession over time.

In the LDA analysis, 4, 3, and 13 taxa in the nodules, rhizosphere soil and bulk soil, respectively, had large effect sizes with LDA score > 2.5. At the genus level, the relative abundances of *Mesorhizobium* and *Bradyrhizobium* had significant differences between AN and SN samples (Figure 3C).

### Association of diazotrophic bacterial communities with environment factors

**Figure 4:**
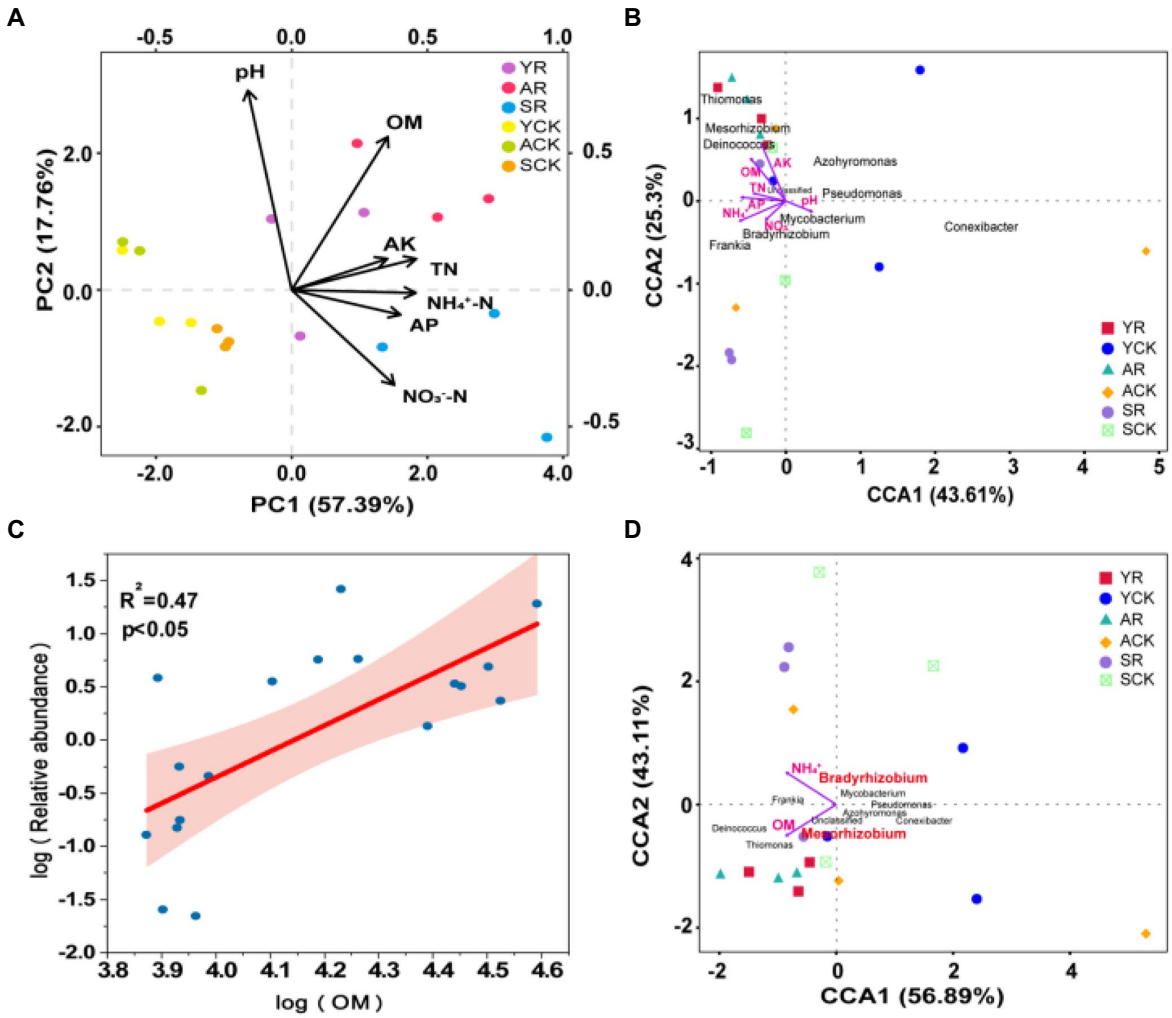
**(A)** PCA analysis based on soil physical and chemical properties. **(B)** CCA analysis of the *nif*H community and soil environmental factors in rhizosphere soil and bulk soil. **(C)** Linear regression relationships between the relative abundance of *Mesorhizobium* and the contents of OM. The relative abundance and contents were log transformed, and shading around regression fits in D are 95% confidence intervals for true fitted values. **(D)** CCA analysis of the *nif*H community and soil environmental factors in rhizosphere soil and bulk soil after BioENV analysis of environmental factors.

PCA analysis based on soil physiochemical properties showed that the samples of rhizosphere soils and bulk soils were clearly separated along axis 2, demonstrating that there was a large environmental heterogeneity between rhizosphere soil and bulk soil. Compared with the bulk soil, the rhizosphere soil samples in the three nodule developing phases were more dispersed, evidencing a great variation in the physicochemical properties of rhizosphere soil with time (Figure 4A).

The pivotal influential factors and their contributions to variations in community composition were performed by CCA. Furthermore, Spearman relationship was used to test the effect of soil physical and chemical properties on the richness of the top 10 species diazotrophic communities at the genus level (Figure 4B). The results showed that AK was significantly positively correlated with *Azohydromonas* (*R*^2^ = 0.377, *p* = 0.007) and *Thiomonas* (*R*^2^ = 0.633, *p* < 0.001), and negatively correlated with *Pseudomonas* (*R*^2^ = 0.220, *p* = 0.050). The OM was significantly positively correlated with *Thiomonas* (*R*^2^ = 0.651, *p* < 0.001) and *Mesorhizobium* (*R*^2^ = 0.347, *p* = 0.010), and the correlation was confirmed by the linear regression relationship between OM and *Mesorhizobium* richness (Figure 4C). The NH_4_^+^-N was significantly positively correlated with *Thiomonas* (*R*^2^ = 0.240, *p* = 0.039) and negatively correlated with *Mycobacterium* (*R*^2^ = 0.272, *p* = 0.026) and *Pseudomonas* (*R*^2^ = 0.376, *p* = 0.007). In addition, BioENV analysis was performed on environmental factors to obtain a combination of environmental factors with the greatest correlation with microbial communities, namely OM and NH_4_^+^-N (Figure 4D). Furthermore, a significant Spearman relationship was observed that the TN, pH, and NO_3_^−^-N were also important factors correlated with the diazotrophic community variation.

### Isolation and identification of rhizobia and their growth characteristics

An increasing number of endophytic bacteria have been isolated recently from the sterilized nodule surfaces of several legumes. In the present study, through preparing root nodule extract in buffer containing antioxidants and cultured in different culture medium, a total of 320 strains of endophytic bacteria were screened from root nodules of *Sophora davidii* in six different counties of northern Shaanxi in hilly and gully regions of the Loess Plateau of China. We showed that the presence of a large diversity of bacteria belonging to 4 phylums, 7 classes, 17 orders, 35 families, and 55 genera, including *Acinetobacter*, *Agrobacterium*, *Agrococcus*, *Agromyces, Arthrobacter*, *Bacillus*, *Brevibacterium*, *Bosea*, *Brachybacterium*, *Brevibacillus*, *Brevundimonas*, *Caulobacter*, *Kaistella*, *Chryseomicrobium*, *Citricoccus*, *Dietzia*, *Exiguobacterium*, *Hymenobacter*, *Isoptericola*, *Lysobacter*, *Massilia*, *Microbacteriaceae*, *Microbacterium*, *Metabacillus*, *Micrococcus*, *Moraxella*, *Mycolicibacterium*, *Nocardia*, *Nocardioide, Ornithinimicrobium*, *Paenarthrobacter*, *Paenibacillus*, *Paeniglutamicibacter*, *Paracoccu, Promicromonospora*, *Pseudarthrobacter, Pseudoclavibacter*, *Pseudoxanthomonas*, *Rathayibacter*, *Rhodococcus*, *Roseomonas*, *Serratia*, *Sphingomonas*, *Staphylococcus*, *Starkeya*, *Stenotrophomonas*, *Streptomyces*, *Variovorax*, *Pseudomonas*, *Phyllobacterium*, *Ochrobactrum*, *Devosia*, *Cupriavidus*, and *Bradyrhizobium*. We also found that the absolutely dominant genus *Mesorhizobium*.

Among them, the bacterial isolates B-26 and BT-30 were identified as *Bradyrhizobium diazoefficiens* and *Mesorhizobium amorphae*, based on the phylogeney of 16S rRNA genes (NCBI accession numbers ON598635 and ON598668, respectively; Supplementary Figure S4). The *nif*H gene sequences of *M. amorphae* BT-30 was matched to OTU_23 (the similarity was 97%), the *nif*H gene sequences of *B. diazoefficiens* B-26 was matched to OTU_8010 (the similarity was 94%).

The utilization profiles of nitrogen and carbon sources of *B. diazoefficiens* B-26 and *M. amorphae* BT-30 were different (Supplementary Figures S5A,B). Both *B. diazoefficiens* B-26 and *M. amorphae* BT-30 could use all the tested N-containing compounds as N sources for growth, but the growth rates were significantly different in most cases for these two strains, even with the same N-source. The optimal nitrogen sources were casein acid hydrolysate, following by yeast extract and Ca(NO_3_)_2_ for *B. diazoefficiens* B-26 and yeast extract following by casein acid hydrolysate and NH_4_Cl for *M. amorphae* BT-30. Both *B. diazoefficiens* B-26 and *M. amorphae* BT-30 could use L-rhamnose, inositol, sucrose, starch soluble, D-glucose and D-mannitol as carbon sources, but the growth of *M. amorphae* BT-30 was always much better than that of *B. diazoefficiens* B-26 utilizing these compounds. Meanwhile, *B. diazoefficiens* B-26 could also use D-arabinose, D-fructose, and D-xylose, but *M. amorphae* BT-30 could not. The optimal nitrogen/carbon sources were casein acid hydrolysate/D-arabinose for *B. diazoefficiens* B-26 and yeast extract/sucrose for *M. amorphae* BT-30. Both strains could grow in medium without addition of NaCl, but sensitive to ≥0.5% of NaCl. The optimal pH was 6 for *M. amorphae* BT-30, and 5–6 for *B. diazoefficiens* BT-26 (Supplementary Figures S5C,D).

### Rhizobia interaction analysis

**Figure 5:**
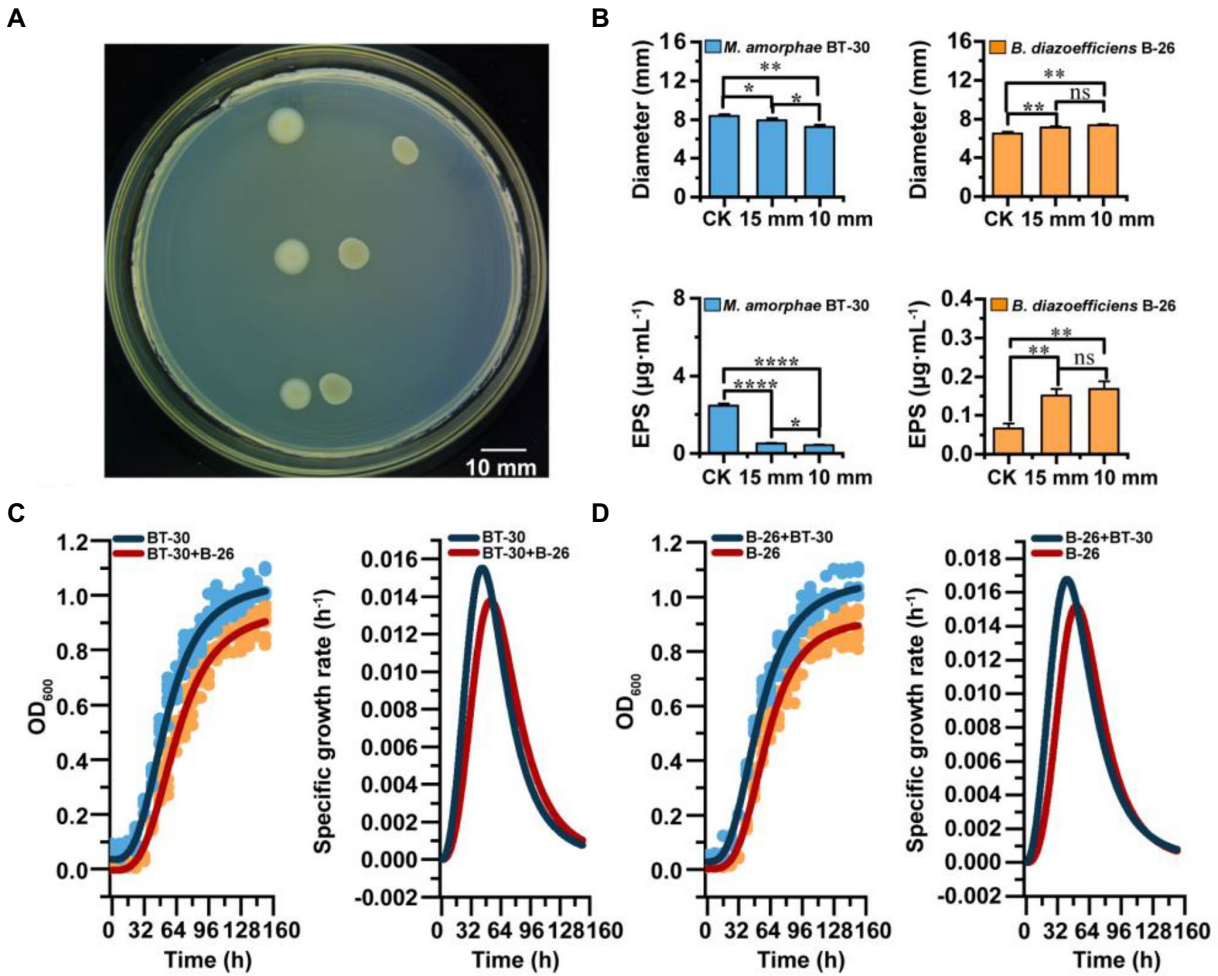
Interaction between *M. amorphae* BT-30 and *B. diazoefficiens* B-26. **(A)** Co-cultivation of *M. amorphae* BT-30 (left) and *B. diazoefficiens* B-26 (right) at different distances (15 mm and 10 mm) on the YMA plate. The control group was at the top, followed by 15 mm and 10 mm distances. **(B)** Colony diameter of *M. amorphae* BT-30 and *B. diazoefficiens* B-26 at 15 mm and 10 mm away from each other on the YMA plate. The production of EPS of *M. amorphae* BT-30 and *B. diazoefficiens* B-26 when they are cocultured on the YMA plate. Asterisks (*, **, *** and ns) indicate significant difference from the control (Welch *t*-test, *p* < 0.05, *p* < 0.01, *p* < 0.001 and not significant). **(C)** The growth curves and specific growth rate curves of *M. amorphae* BT-30 under co-culture and pure culture conditions. **(D)** The growth curves and specific growth rate curves of *B. diazoefficiens* B-26 under co-culture and pure culture conditions.

When *B. diazoefficiens* B-26 and *M. amorphae* BT-30 were co-cultured on YMA medium for 6 days, the colony diameters (7.339 ± 0.130 mm) and EPS production (0.168 ± 0.020 μg·ml^−1^) of *B. diazoefficiens* B-26 adjacent to *M. amorphae* BT-30 (at a 10 mm and 15 mm distance) were significantly greater than that of the control (6.481 ± 0.194 mm, 0.066 ± 0.012 μg·ml^−1^; Figure 5). While the growth and EPS production of *M. amorphae* BT-30 adjacent to *B. diazoefficiens* B-26 (10 mm) were significantly inhibited, with decreased colony diameters/EPS along the reducing of distance (Figures 5A,B).

Fitted the growth curve of strains by Logistic regression model (Supplementary Table S4), the growth curves and specific growth rate curves illustrated that growth of *M. amorphae* BT-30 was inhibited by *B. diazoefficiens* B-26, since both the growth rate and specific growth rate of BT-30 were lower in co-culture than that in the monoculture (Figure 5C). Meanwhile, growth of *B. diazoefficiens* B-26 was promoted by *M. amorphae* BT-30, since both the growth rate and specific growth rate of B-26 were greater in co-culture than that in the monoculture (Figure 5D).

## Discussion

Legumes are known as pioneer plants and enhancers of the nutritional status in cultivated soils and barren land, which has been explained by their capacity to form symbiosis with rhizobia, since they give an indication of the host specificity and nitrogen fixing ability (Diouf et al., 2010). Moreover, most of the previous studies have mainly involved analysis of the bacterial diversity of rhizobia and legume plant-microbe interactions, while little systemic observation has been paid to the interactions between different rhizobia within the root nodules. To date there has been no evaluation of the structure dynamics of diazotrophic communities in relation to the development of root nodules in the pioneer legumes. As a pioneer plant, *S. davidii* widely grows in poor soils of the Loess Plateau of China, for which *Mesorhizobium* sp. X, *M. waimense*, and *M. amorphae* have been reported as the dominant and universal symbionts in the forest area of the Loess Plateau in northern Shaanxi Province (Cao et al., 2021). In the present study, we used *S. davidii* as a model plant for investigating the dynamics of diazotrophic communities in the root nodules.

Nitrogenase is essential in BNF, it is a complex of proteins encoded by the *nif*H, *nif*D, and *nif*K genes (Rubio and Ludden, 2002), in which *nif*H has been widely used for investigating the diversity and composition of diazotroph communities. Due to the fact that *nif*H is a highly conserved gene (Gaby and Buckley, 2014), it is a suitable molecular biomarker to detect the diazotrophic microbes (Collavino et al., 2014). In our present study, high-throughput *nif*H amplicon sequencing was performed on the nodules and soils associating with *S. davidii,* across different nodule developing phases to explore the succession of diazotrophic communities. Our results revealed that the root nodules of *S. davidii* were occupied by diverse bacteria dominated by *Mesorhizobium* (96.579%–99.734%), implying that they were the microsymbionts of this host plant as reported previously (Cao et al., 2021). However, other diazotrophs including *Bradyrhizobium* (0.071%–2.806%) were also detected inside the nodules of *S. davidii*. It is not clear if these endophytic diazotrophs, especially *Bradyrhizobium,* also symbionts for this legume. Previously, *Bradyrhizobium* has been reported as microsymbionts for diverse legumes (Ormeño-Orrillo and Martínez-Romero, 2019), including *Sophora flavescens* (Wu et al., 2021). Anyway, the minor groups of the diazotrophs can be nonsymbiotic nodule endophytes since this kind bacteria have been frequently isolated from nodules of diverse legumes (Deng et al., 2020; Geetha Thanuja et al., 2020).

Previous studies found that the microbial community composition of the bulk soil and the rhizosphere environment compartments are distinct (Reinhold-Hurek et al., 2015), and its diversity decreases with proximity to the root in relation to the plant selection (Bulgarelli et al., 2013; Donn et al., 2015). The results for the diazotrops investigated in our present study (Figure 2) was consistent with the previous studies, e.g., all the diversity indices were significantly lower in nodule endosphere than those in rhizosphere and bulk soils. Meanwhile, the abundances of *Mesorhizobium* were increased and that of *Bradyrhizobium* were decreased in the young and active phases, evidencing that *Mesorhizobium*, but not *Bradyrhizobium*, was strongly selected by the nodules as the microsymbiont for this host. Furthermore, clear succession in the diazotrophic community was observed in all the three studied compartments: nodule endosphere, rhizosphere soil and bulk soil, remarkable evidenced by the variation of *Bradyrhizobium* (Figure 3). Previous studies have shown that bacterial diversity and abundance (Reed et al., 2010) associated with N-fixation rates. For example, the diazotrophic community composition of biocrusts in semiarid grassland undergoes strong seasonal shifts and the abundance of its dominant members decreased in response to more frequent with small volume precipitation events (Yeager et al., 2012).

In all the three compartments, the relative abundance of *Bradyrhizobium* was significantly increased in the senescent phase, demonstrating that this bacterium was greatly benefited by the senescence of nodules. In its senescent phase, the nodules lost its ability of BNF and presented high proteinase activity (Van de Velde et al., 2006), implying the possibility to release more NH_4_^+^ and organic C inside and outside the nodules. So, the increase of *Bradyrhizobium* in the senescent phase might imply that *Bradyrhizobium* lives as saprophyte (Hansen et al., 2020) in all the three studied compartments associated with *S. davidii* plants. The saprophytic life style of *Bradyrhizobium* was confirmed by the increased contents of NH_4_^+^-N and TN in rhizosphere soil and bulk soil in the senescent phase (Supplementary Figure S1), and by its positive correlation with NH_4_^+^-N and TN in CCA analysis (Figure 4). However, why *Bradyrhizobium*, but not other diazotrophs, obtained significant benefits from senescence of *S. davidii* nodules is not clear.

Our results in the present study also demonstrated that the succession of diazotrophs along the nodule development was represented mainly by the variation in relative abundance of *Bradyrhizobium* in all the three compartments (Figure 3). In nodules, ratio of *Mesorhizobium* and *Bradyrhizobium* seemed the main factor of the succession, which might be controlled by the interaction between the symbionts and the host plants (Figure 3), since *S. davidii* specifically formed nodules with *Mesorhizobium* species (Cao et al., 2021). In another hand, in rhizosphere and bulk soils, the succession of diazotrophic communities along with the nodule development was mainly affected by environmental factors, such as nutrients and pH values. The ratio of positive and negative interactions among the bacteria depends on the nutrients in the environment (Ratzke et al., 2020), with more nutrients allowing for more bacteria to grow in monoculture, which then leads to more competition (Kehe et al., 2019).

Compared with bulk soil, rhizosphere significantly enriched *Mesorhizobium* in young and active phases (Figure 3). The positive correlation of *Mesorhizobium* with OM and AK (Figure 4). The abundance decreased of *Bradyrhizobium* in rhizosphere than that in bulk soil in the young and active phases might be related to its competence for nutrients with the increased *Mesorhizobium*. Compared with that in the young and active phases of nodules, *Bradyrhizobium* in senescent phase was much greater in both the rhizosphere and bulk soils, and also enriched in rhizosphere than in bulk soil, suggesting again the nodule senescence specifically stimulate *Bradyrhizobium* by root exudation. This is consistent with the previous observation that different bacteria were specified in different niches and host plants actively filtrated bacteria by exudates (Bulgarelli et al., 2013; Yuan et al., 2021).

Our findings show that microbial communities in three compartments were associated with various edaphic physical and chemical properties variables, and compared with *M. amorphae* BT-30, *B. diazoefficiens* B-26 was more suitable for acidic environment, which could explain their significant difference in biogeographic patterns among the three compartments. Plant played important roles in determining the community composition and structure of the rhizospheric microbiome though recruiting specific microbiomes (Berendsen et al., 2012), these findings suggest that the alpha diversity with root proximity showed a decreasing trend owing to the root filtration and selection effects (Dennis et al., 2010).

The existence of a large number of species makes the *in situ* interactions among species very complex and requires a simplified system to dissect the dynamic behavior of populations. Previous studies revealed that community changes could be largely explained by binary interactions (Schäfer et al., 2022), indicating that assembly rules based on pairwise interactions (Friedman et al., 2017). Following these findings, in the present study, we selected two strains of *M. amorphae* BT-30 and *B. diazoefficiens* B-26 and further focused on a prominent interaction between two members, to test whether two species undergo ecological cooperation or competition upon co-cultured conditions on artificial medium (YMA plate).

In the coculture experiment, we found that the strains *M. amorphae* BT-30 and *B. diazoefficiens* B-26 showed a competitive relationship under co-culture conditions, in which *B. diazoefficiens* B-26 significantly inhibited the growth of *M. amorphae* BT-30 (Figure 5). This result parallels observations made in the pairwise experiments (Ortiz et al., 2021; Weiss et al., 2022). The phenotype analysis revealed that *B. diazoefficiens* B-26 and *M. amorphae* BT-30 could utilize some common carbon and nitrogen sources, but their growth (OD_600_) could be very different (Supplementary Figure S5). Study shown that the rhizosphere microbiome assembles from pioneer assemblages of species with random resource overlap into high-density, functionally complementary climax communities at later stages (Hu et al., 2020). This also explained why the relative abundance of *Bradyrhizobium* increases significantly during the senescent phase. Thus, niche differentiation about the terms of resource use patterns played an important role in nodule-associated diazotrophic community succession. Furthermore *B. diazoefficiens* B-26 presented a wider range of carbon sources and pH range than *M. amorphae* BT-30, especially at alkaline condition (pH8.5), which might be explain why *Bradyrhizobium* was much more abundant than *Mesorhizobium* in bulk soil, where presented pH >8 (Supplementary Figure S1).

Overall, our results indicate that root nodules have a microbial community that is dynamic across root nodule developmental phases. Additionally, we also observed that the *M. amorphae* BT-30 may specifically promote growth of *B. diazoefficiens* B-26, while *B. diazoefficiens* B-26 inhibit the growth of *M. amorphae* BT-30. And the two are complementary in the use of carbon and nitrogen sources. This led to the key finding that developmental phases of nodules and niche between different diazotrophic communities were the key driving factors for the succession of nodule-associated diazotrophic community.

## Conclusion

In this study, the spatial (microhabitat) and temporal (three developmental phases of the root nodule) of diazotrophic communities’ succession were extensively summarized by high-throughput *nif*H amplicon sequencing. Our results demonstrate that there is a discrepancy between the α-diversity and composition of diazotrophic communities in different microhabitat and phases. The microbial community composition was distinct different in root nodules, rhizosphere and bulk soil, indicating the environmental filtering differed in the assembly of bacterial communities. Beyond this, our results demonstrated the presence of diverse diazotrophic bacteria and successional community shifting dominated by *Mesorhizobium* and *Bradyrhizobium* inside the nodule according to the nodule development. The relative abundance decreased for *Mesorhizobium*, while decreased first and then increased for *Bradyrhizobium* in nodule development from young to active to senescent. Collectively, our study highlights that developmental phases of nodules combined with microhabitat of *Sophora davidii* were the key driving factors for the succession of nodule-associated diazotrophic community.

## Data availability statement

The datasets presented in this study can be found in online repositories. The names of the repository/repositories and accession number(s) can be found in the article/Supplementary material.

## Author contributions

JA: experimentation and writing–original draft. TY: data curation and software. XL: conceptualization and methodology. YJ: conceptualization and methodology. ZH and XZ: experimentation. EW: writing–review and editing. ZD: methodology, project administration, and writing–review and editing. All authors contributed to the article and approved the submitted version.

## Funding

This work was supported by the National Natural Science Foundation of China (grant no. 32160003).

## Conflict of interest

The authors declare that the research was conducted in the absence of any commercial or financial relationships that could be construed as a potential conflict of interest.

## Publisher’s note

All claims expressed in this article are solely those of the authors and do not necessarily represent those of their affiliated organizations, or those of the publisher, the editors and the reviewers. Any product that may be evaluated in this article, or claim that may be made by its manufacturer, is not guaranteed or endorsed by the publisher.
