# Supplementary figures and images for: Nodule-associated diazotrophic community succession is driven by developmental phases combined with microhabitat of Sophora davidii
Source: Front Microbiol. 2022 Dec 1;13:1078208. doi: 10.3389/fmicb.2022.1078208 (PMC9751200; doi:10.3389/fmicb.2022.1078208)

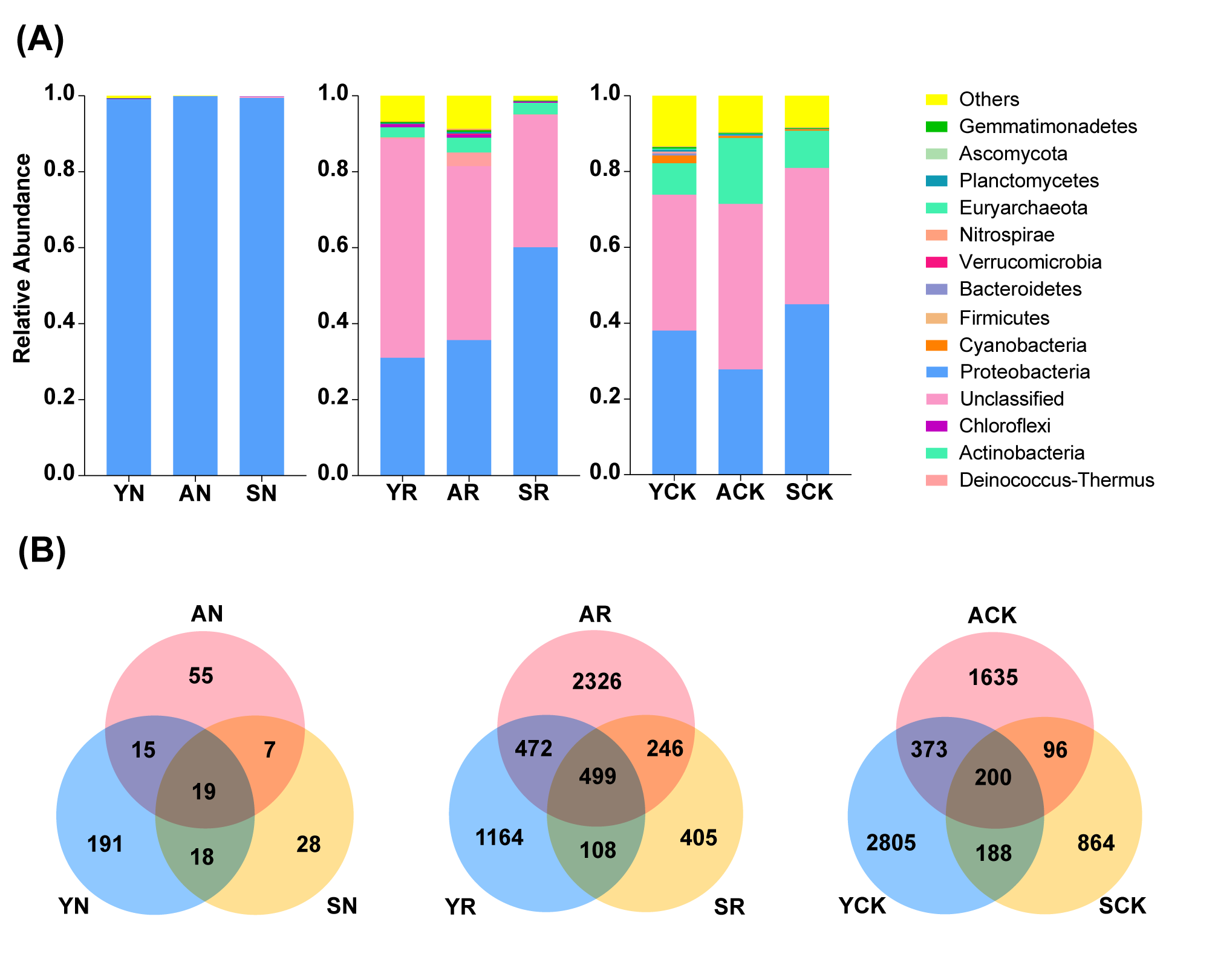

Supplement: Supplementary file 1 [file Image_1.TIF]

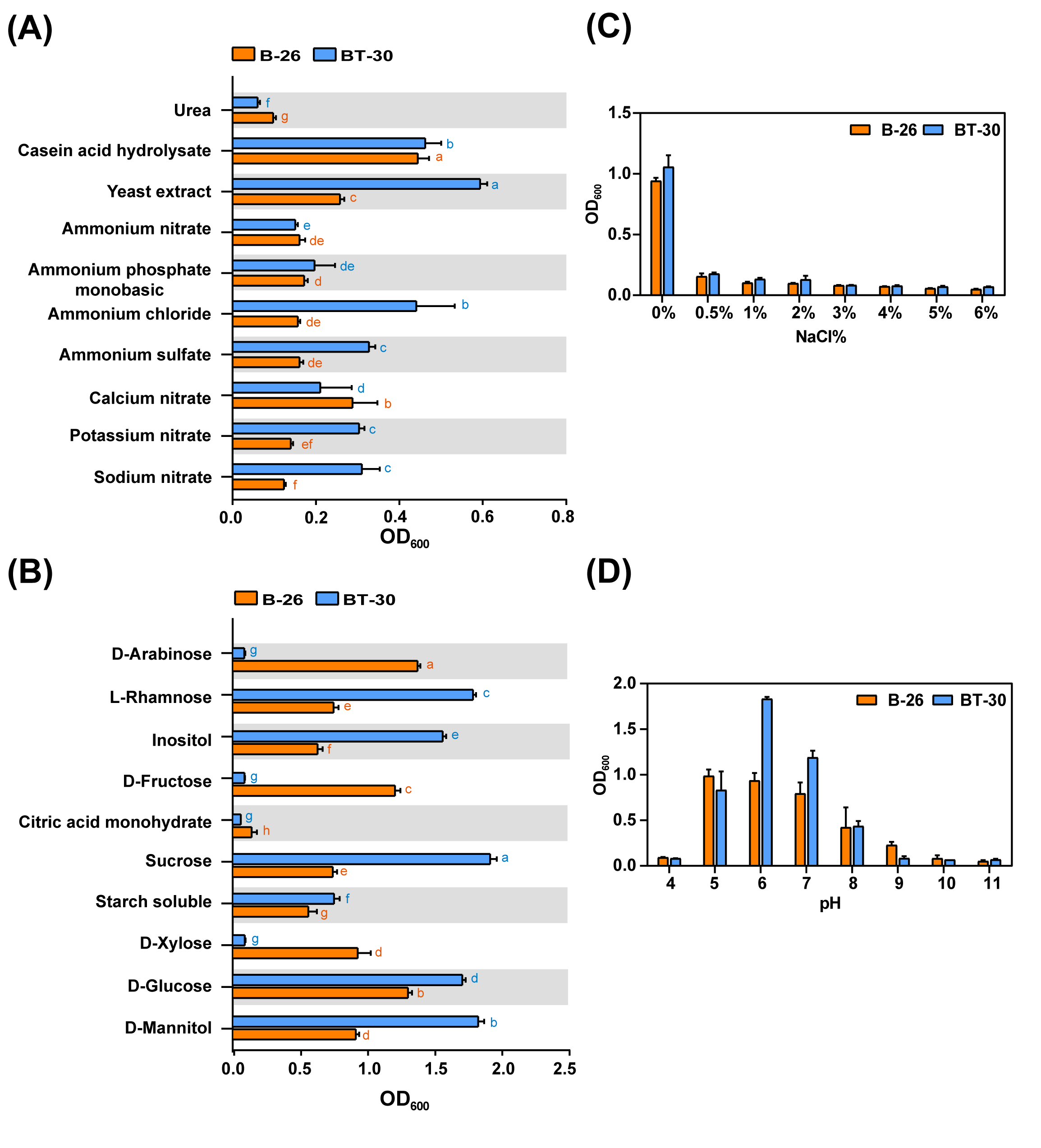

Supplement: Supplementary file 2 [file Image_2.TIF]

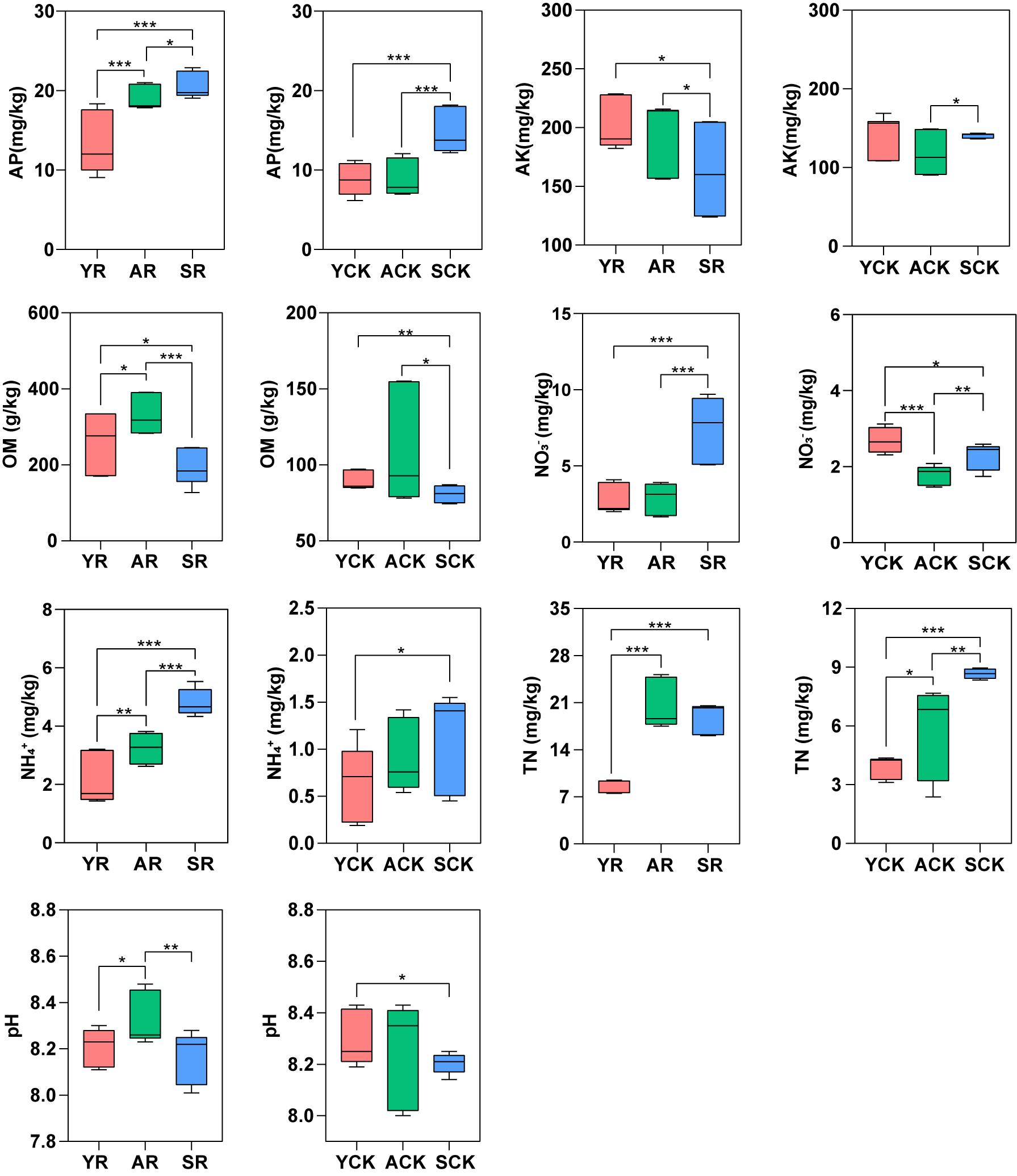

Supplement: Supplementary file 3 [file Image_3.TIF]

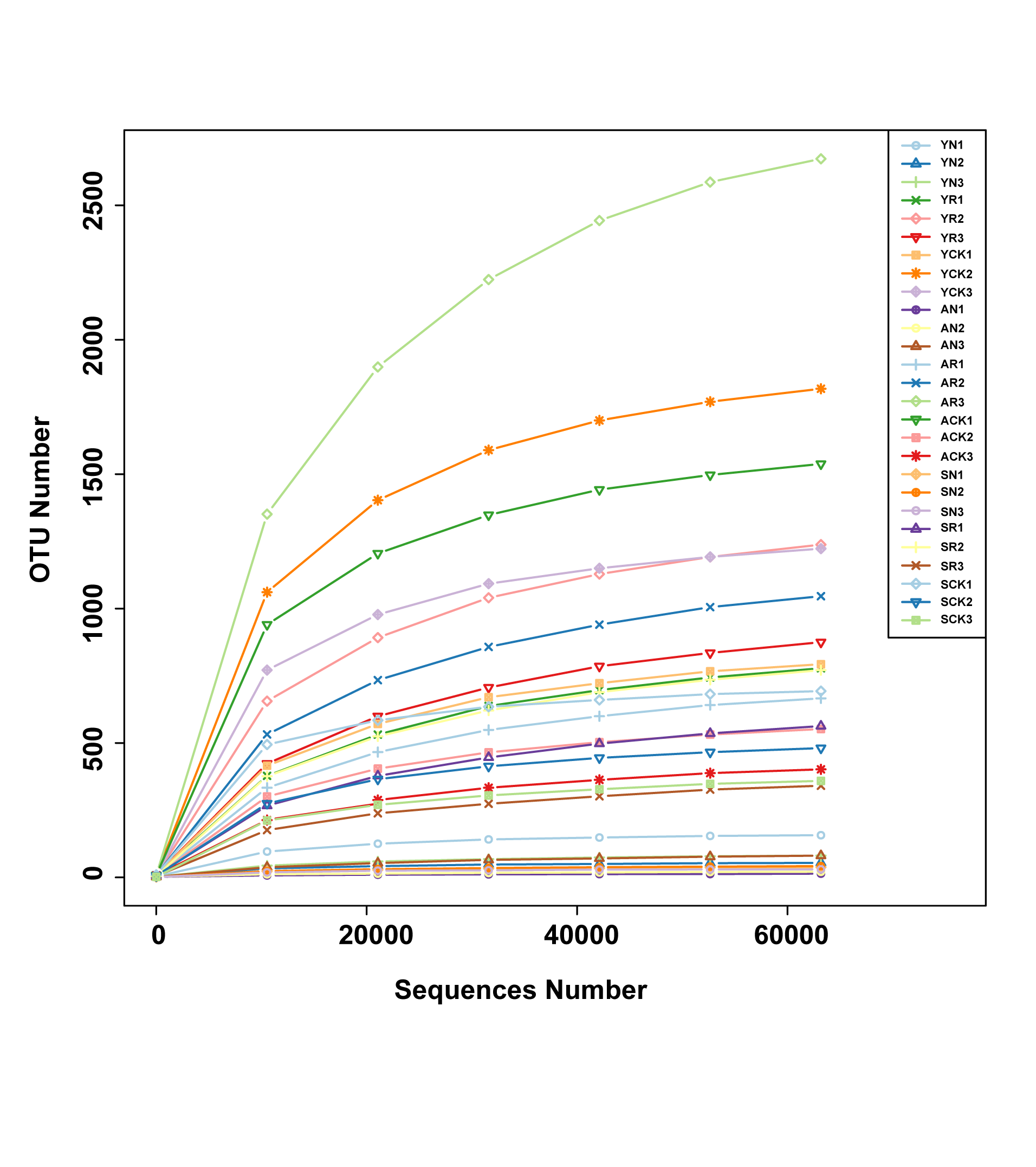

Supplement: Supplementary file 4 [file Image_4.TIF]

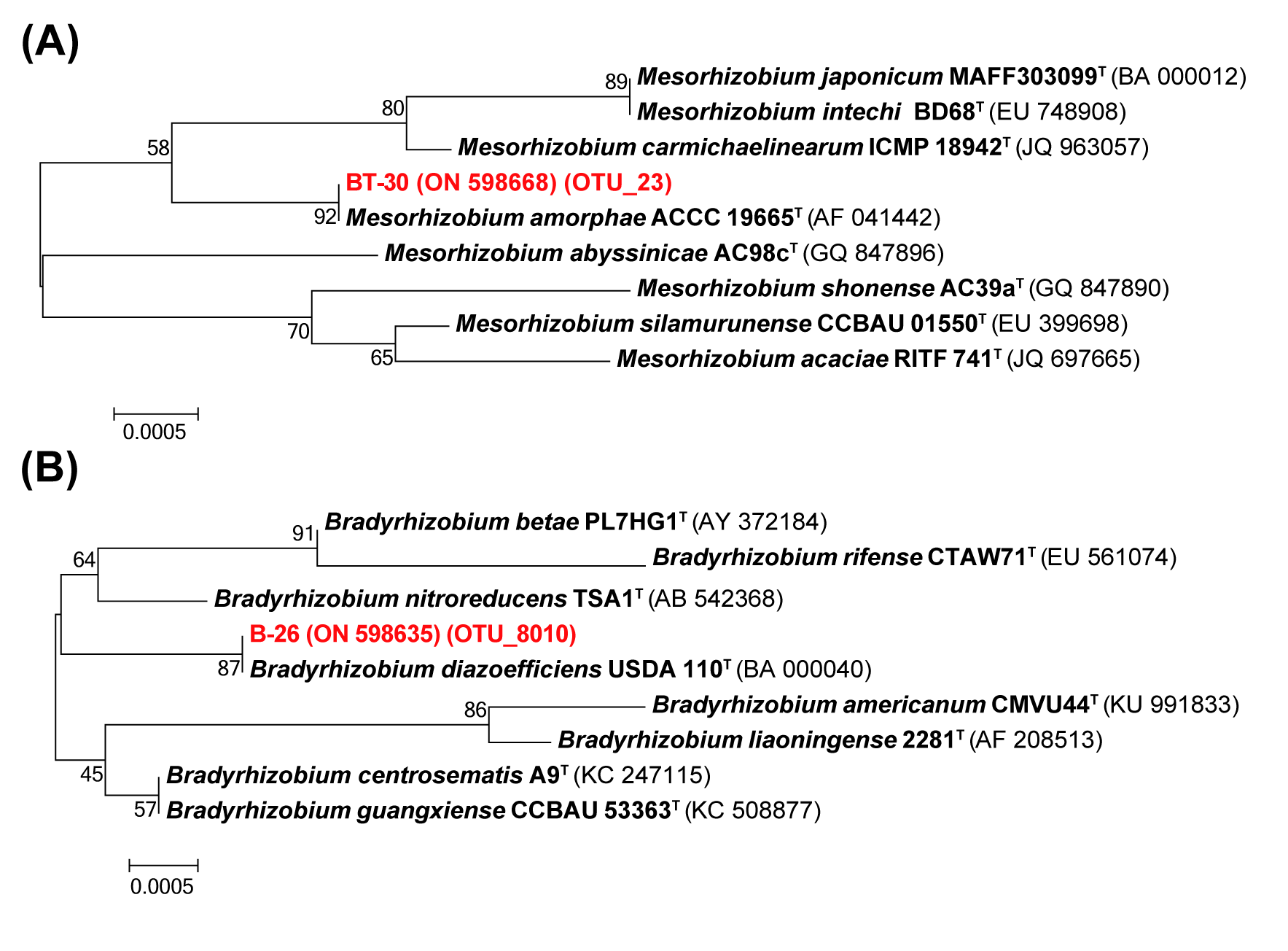

Supplement: Supplementary file 5 [file Image_5.TIF]
