# Supplementary material for: Nodule-associated diazotrophic community succession is driven by developmental phases combined with microhabitat of Sophora davidii
Source: Front Microbiol. 2022 Dec 1;13:1078208. doi: 10.3389/fmicb.2022.1078208 (PMC9751200; doi:10.3389/fmicb.2022.1078208)
Supplement: Supplementary file 6 [file Data_Sheet_1.docx]

**Table S1. Methods used to analyze the soil chemical properties.**

| Soil chemical properties | Methods (references) | Analytical instruments (type,manufacturer) |
| --- | --- | --- |
| Total nitrogen (TN) | Kjeldahl method | Ultraviolet spectral photometer  (UV-1780, Shimadu, Tokyo, Japan) |
| Available phosphorus (AP) | 0.5 M NaHCO3 extraction (Wang et al., 2020) | Ultraviolet spectral photometer  (UV-1780, Shimadu, Tokyo, Japan) |
| Available potassium (AK) | 1 M NH4OAC extraction | Flame photometer  (FP640, YiDian, Shanghai, China) |
| Total organic matter (OM) | K2Cr2O4 volumetric method (Chen et al., 2015) | Oil bath K_2_CrO_7_ titration method |
| pH | 1:2.5 water-soluble extract | pH meter  (PHS-3E, YiDian, Shanghai, China) |
| Nitrate nitrogen (NO_3_^-^-N) | Colorimetry (Liu et al., 2020) | Ultraviolet spectral photometer  (UV-1780, Shimadu, Tokyo, Japan) |
| Ammonium nitrogen（NH_4_^+^-N） | Nessler’s reagent (420 nm) (Liu et al., 2020) | Ultraviolet spectral photometer  (UV-1780, Shimadu, Tokyo, Japan) |

**Table S2.** T-text results of relative abundance of *Mesorhizobium* between different samples (*p* value)

| Samples | Young phase | Active phase | Senescent phase |
| --- | --- | --- | --- |
| Nodule vs Rhizosphere | 0.00754 | 0.00308 | 0.00004 |
| Nodule vs Bulk soil | 0.00003 | 0.00027 | 0.00056 |
| Rhizosphere vs Bulk soil | 0.31863 | 0.36543 | 0.11225 |

**Table S3.** T-text results of relative abundance of *Bradyrhizobium* between different samples (*p* value)

| Samples | Young phase | Active phase | Senescent phase |
| --- | --- | --- | --- |
| Nodule vs Rhizosphere | 0.04690 | 0.00940 | 0.03890 |
| Nodule vs Bulk soil | 0.25564 | 0.41918 | 0.33917 |
| Rhizosphere vs Bulk soil | 0.76329 | 0.89021 | 0.58775 |

**Table S4.** **Fitting parameters of strain growth curve by Logistic regression model**

|  | BT-30 | BT-30+B-26 | B-26 | B-26+BT-30 |
| --- | --- | --- | --- | --- |
| Model | Logistic | | | |
| Equation | y = A2 + (A1-A2) / (1 + (x/x0) ^p) | | | |
| A1 | 0.02901 ± 0.0142 | 0.00263 ± 0.01322 | -0.00398 ± 0.01095 | 0.0742 ± 0.01397 |
| A2 | 1.07094 ± 0.01872 | 0.92522 ± 0.01855 | 0.9528 ± 0.01938 | 1.05433 ± 0.01927 |
| x0 | 51.99641 ± 1.10346 | 59.27042 ± 1.21849 | 63.49898 ± 1.20654 | 54.23515 ± 1.20135 |
| R-square (COD) | 0.99695 | 0.99608 | 0.99737 | 0.99653 |
| Adjusted R-square | 0.99638 | 0.99534 | 0.99687 | 0.99587 |

Chen, Z., Wu, W., Shao, X., Li, L., Guo, Y., Ding, G., 2015. Shifts in Abundance and Diversity of Soil Ammonia-Oxidizing Bacteria and Archaea Associated with Land Restoration in a Semi-Arid Ecosystem. PloS one 10, e0132879.

Liu, Z., Li, D., Zhang, J., Saleem, M., Zhang, Y., Ma, R., He, Y., Yang, J., Xiang, H., Wei, H., 2020. Effect of simulated acid rain on soil CO2, CH4 and N2O emissions and microbial communities in an agricultural soil. Geoderma 366, 114222.

Wang, X., Zhang, Z., Yu, Z., Shen, G., Cheng, H., Tao, S., 2020. Composition and diversity of soil microbial communities in the alpine wetland and alpine forest ecosystems on the Tibetan Plateau. Science of The Total Environment 747, 141358.
