# Supplementary material for: Nodule-associated diazotrophic community succession is driven by developmental phases combined with microhabitat of Sophora davidii
Source: Front Microbiol. 2022 Dec 1;13:1078208. doi: 10.3389/fmicb.2022.1078208 (PMC9751200; doi:10.3389/fmicb.2022.1078208)
Supplement: Supplementary file 7 [file Data_Sheet_2.docx]

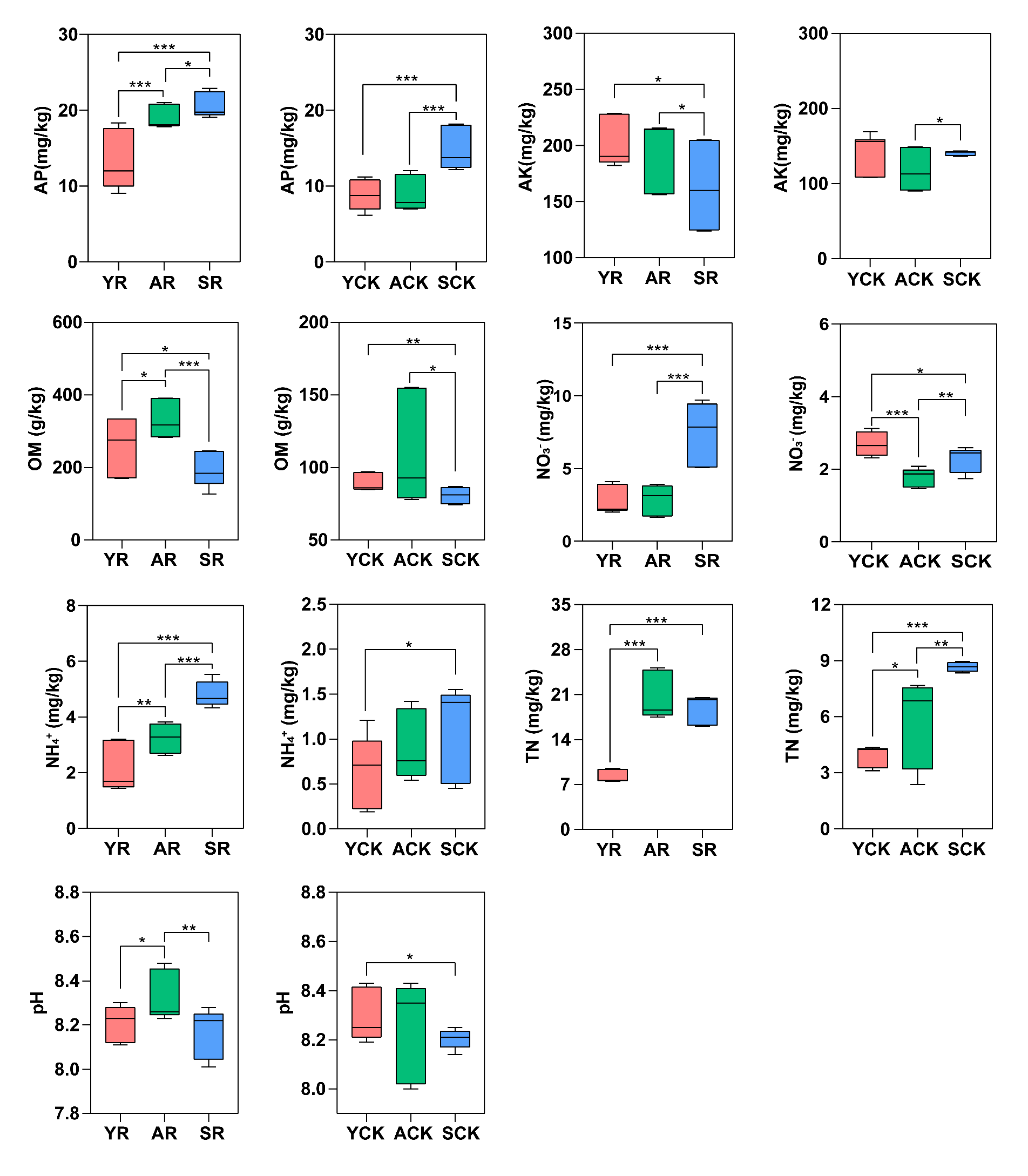

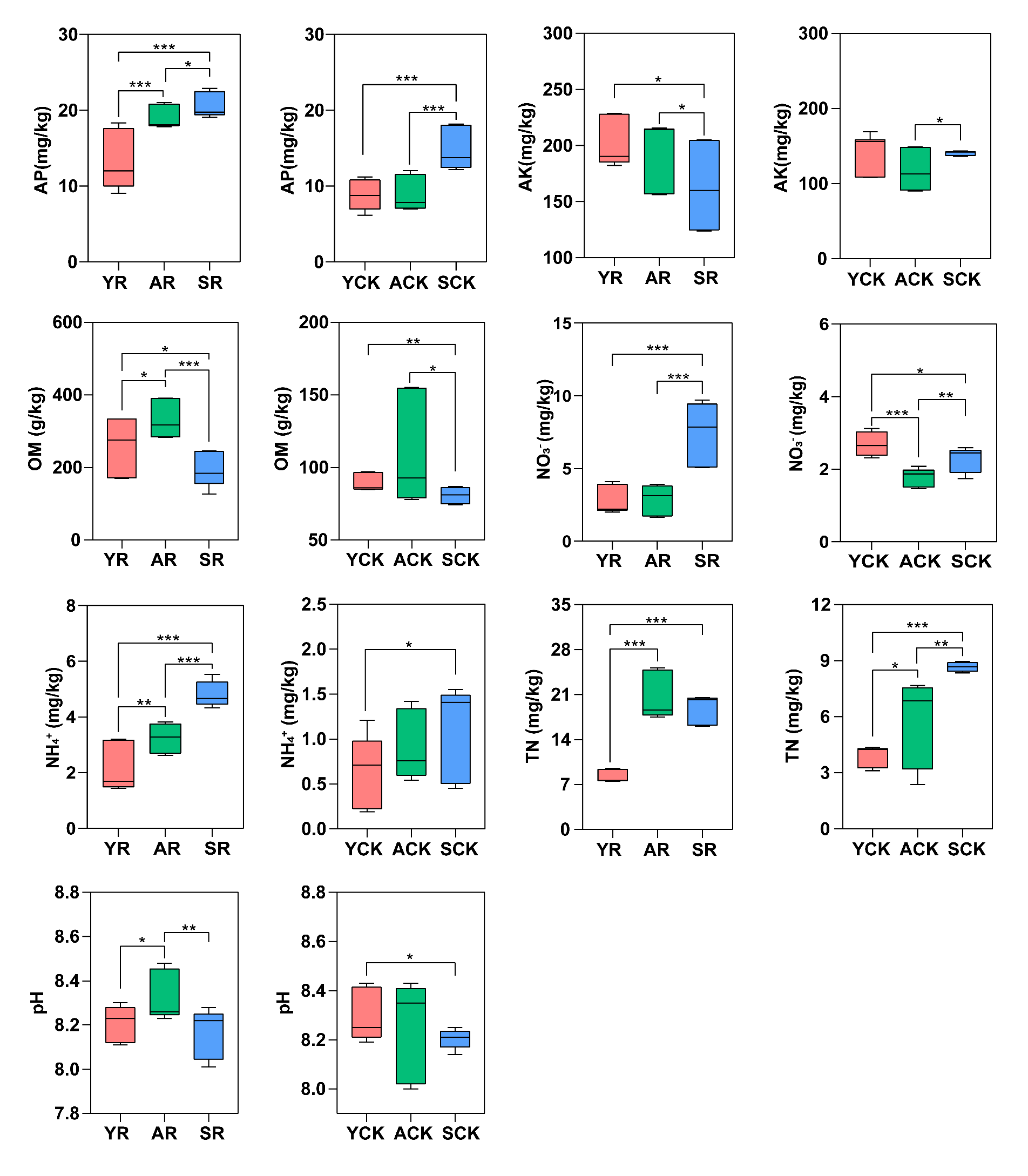


**Supplementary Figure 1.** Soil physiochemical factors of sampling sites. Letters Y, A, and S correspond to young, active and senescent phases of nodules, respectively; R and CK represent rhizosphere and bulk soil, respectively.


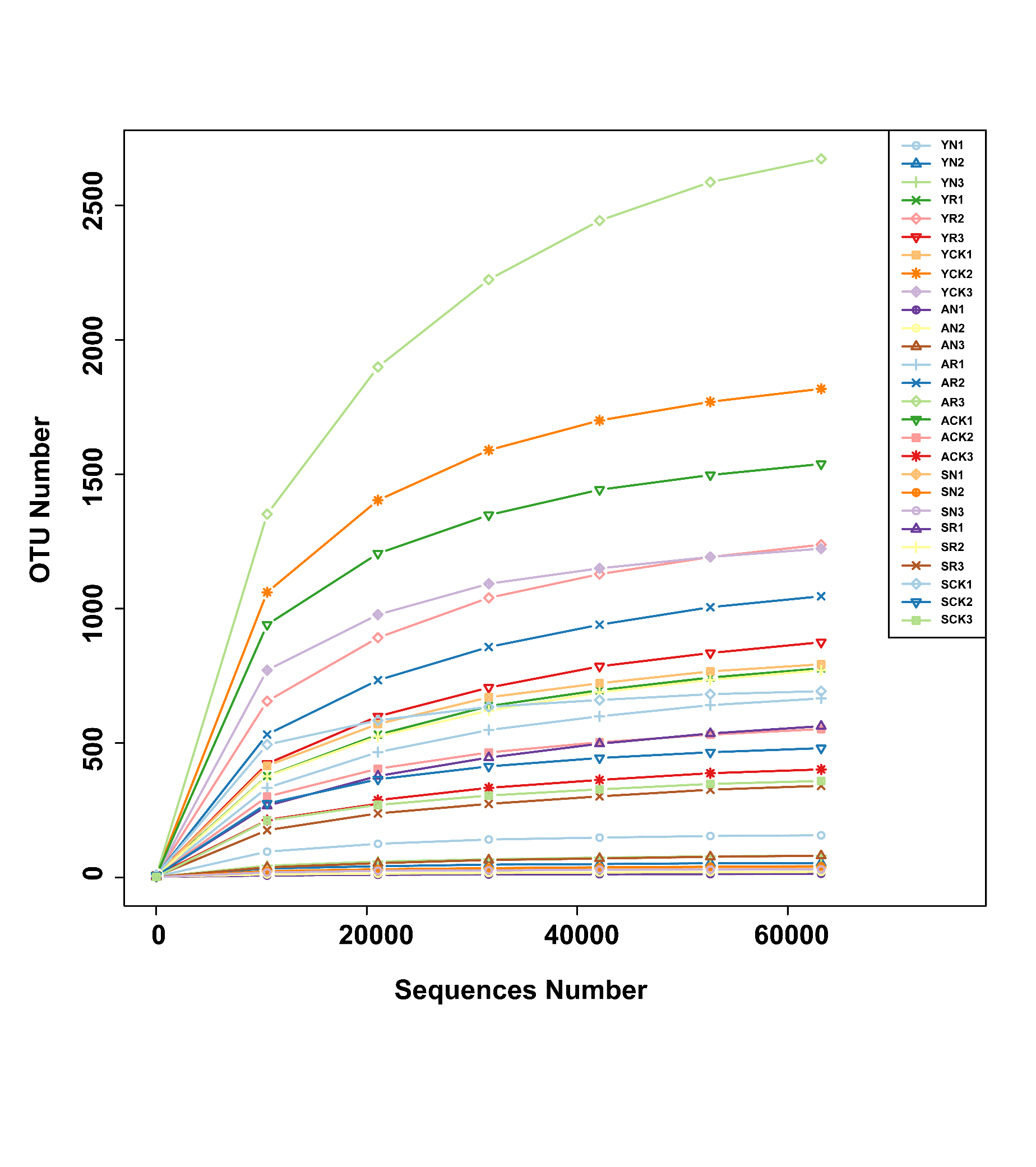


**Supplementary Figure 2.** Rarefaction curves of soil, rhizosphere and nodule samples.


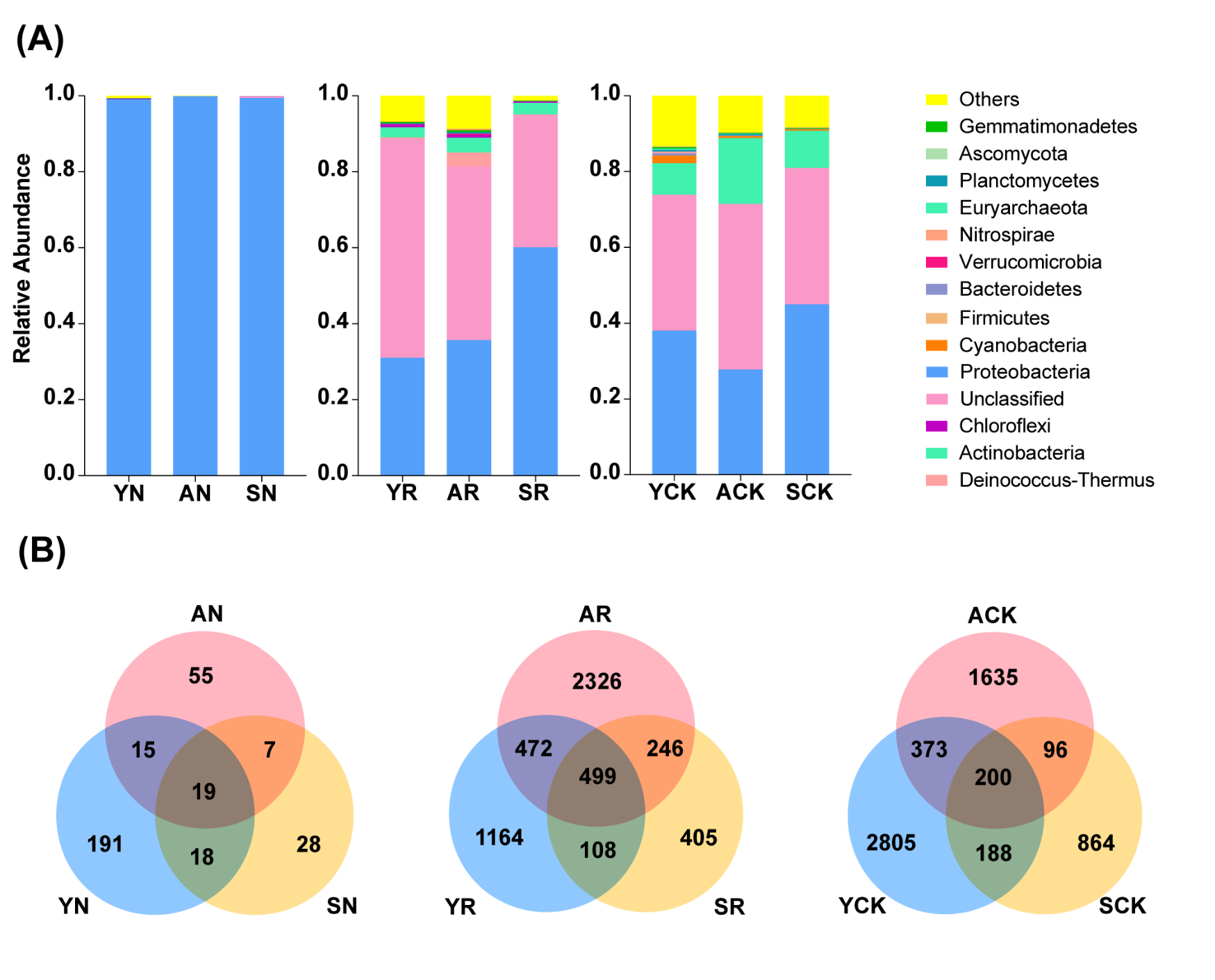


**Supplementary Figure 3.** A. Distribution of the nitrogen-fixing microorganism across in different samples at phylum level. B. Venn diagrams showing the distribution of the nitrogen-fixing microorganism genera across in different samples.


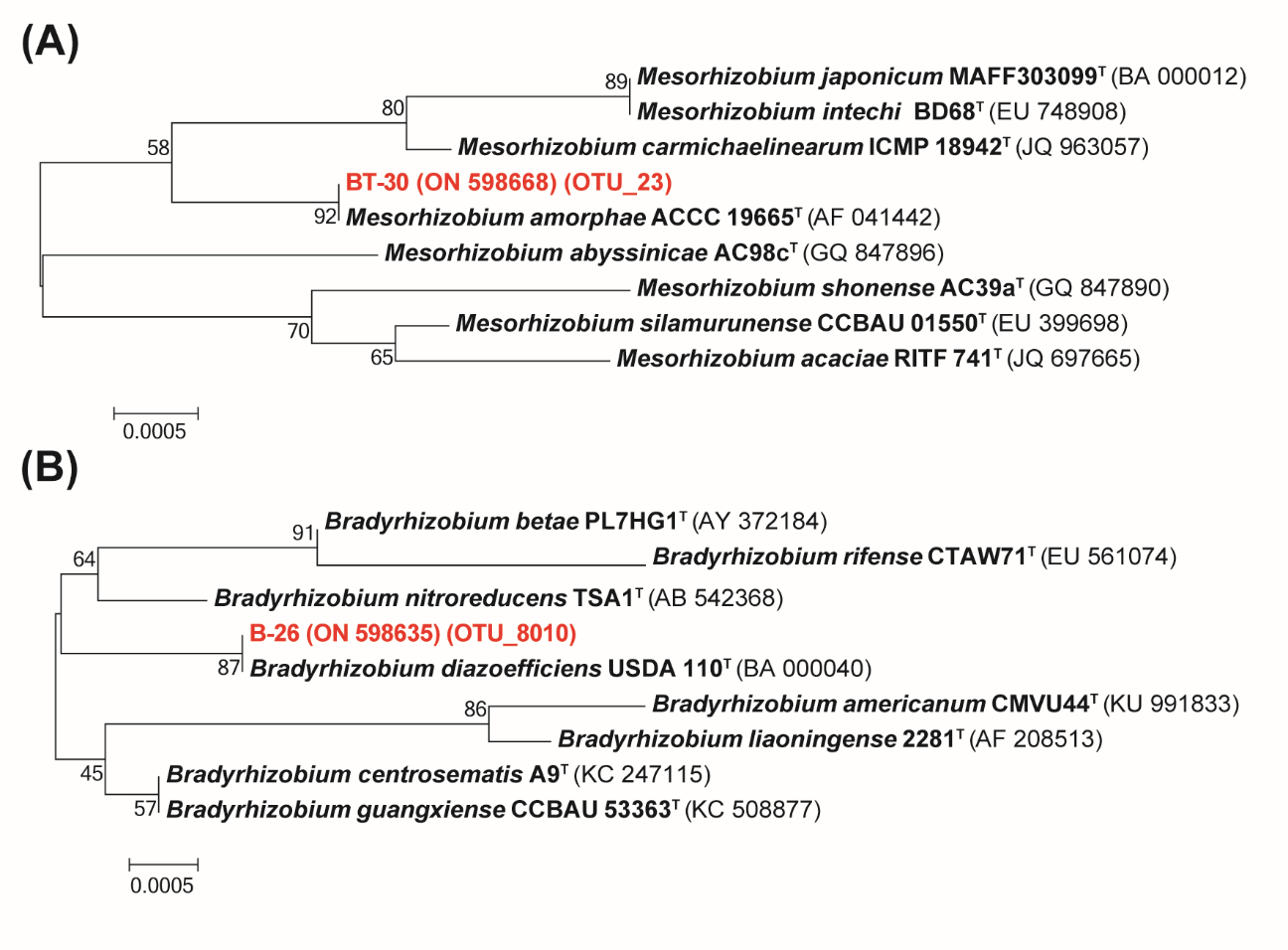


**Supplementary Figure 4.** A. The phylogenetic tree of *M. amorphae* BT-30 based on 16S rRNA gene sequence. A. The phylogenetic tree of *B. diazoefficiens* B-26 based on 16S rRNA gene sequence.


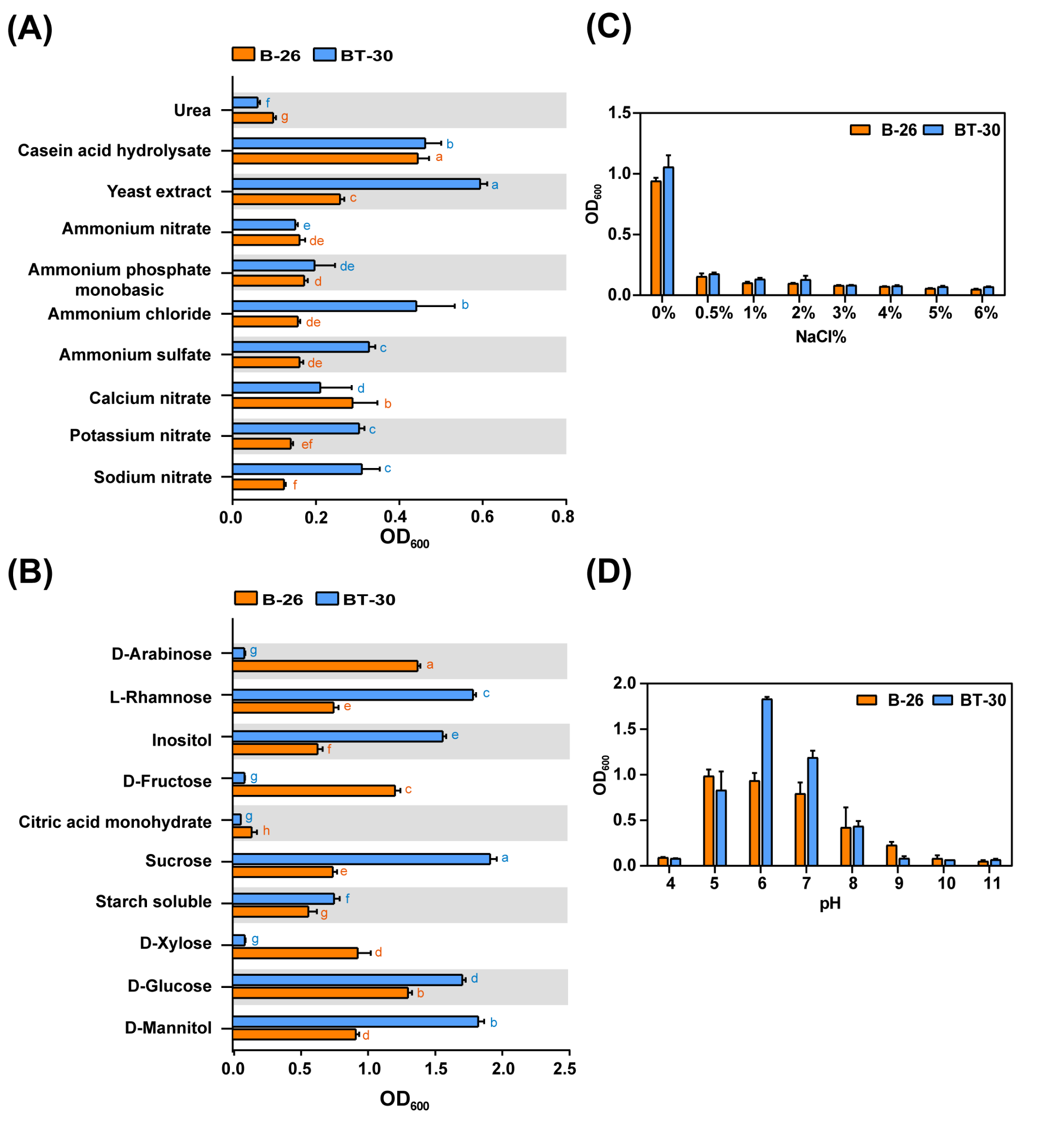


**Supplementary Figure 5.** Growth of *M. amorphae* BT-30 and *B. diazoefficiens* B-26 under different conditions. A. different nitrogen sources. B. different carbon sources. C. different salt concentrations. D different pH conditions.
